# Classification and Segmentation of Medical Images Using Cross-Representation Attention Fusion and Fuzzy Image Enhancement

**DOI:** 10.3390/s26144364

**Published:** 2026-07-09

**Authors:** Abror Shavkatovich Buriboev, Ryumduck Oh, Nishanov Akhram, Khurshid Dusonov, Inomjon Narzullaev, Shavkat Buribayev, Ozod Yusupov, Abbos Abduvaytov, Aziza Axmedova, Cheolwon Lee, Heung Seok Jeon

**Affiliations:** 1 Department of Artificial Intelligence, Gachon University, Seongnam 13120, Republic of Korea; abror1989@gachon.ac.kr; 2 Department of Computer Engineering, Korea National University of Transportation, Chungju 27469, Republic of Korea; rdoh@ut.ac.kr; 3 Department of Software of Information Technologies, Tashkent University of Information Technologies Named After Muhammad Al-Khwarizmi, Tashkent 100084, Uzbekistan; nishanovakhram@gmail.com (N.A.); xurshiddusanov@gmail.com (K.D.); i.narzullayev@tuit.uz (I.N.); 4 Department of CE, Samarkand State Technical University, Samarkand 140143, Uzbekistan; abbosshav@gmail.com; 5 Department of Software Engineering, Samarkand State University, Samarkand 140104, Uzbekistan; ozodyusupov@gmail.com; 6 Department of IT, Samarkand Institute of Economy and Service, Samarkand 140100, Uzbekistan; abbosabduvaytov069@gmail.com; 7 Department of Exact Sciences, Kimyo International University in Tashkent, Tashkent 100121, Uzbekistan; aziza_axmedova@kiut.uz; 8 Department of Computer Engineering, Konkuk University, Chungju 27478, Republic of Korea; 9 AI Convergence Research Center, Konkuk University, Chungju 27478, Republic of Korea

**Keywords:** image analysis, deep learning, segmentation, chest X-ray, renal imaging, fuzzy image enhancement, cross-representation attention, multi-task learning, classification, and lesion delineation

## Abstract

This paper proposes a Cross-Representation Attention-Based Neural Network with fuzzy image enhancement for joint classification and segmentation of chest X-ray and kidney images. First, each input image is transformed into three complementary representations using histogram spread, fuzzy entropy, and fuzzy standard deviation-based enhancement. These representations emphasize different intensity distributions, informative regions, and local structural variations. A Cross-Representation Attention Fusion module then models multidirectional relationships among the enhanced representations and adaptively integrates their complementary features into a unified feature space. The fused features are processed by a shared encoder with task-specific classification and segmentation heads. The framework is evaluated for clinically relevant chest X-ray abnormalities, including pneumonia, pneumothorax, pleural effusion, and lung opacity, and for kidney-image classes comprising normal, tumor/renal cell carcinoma, and cystic renal mass cases. Experimental results show that the proposed method outperforms conventional and recent baseline models in both classification and segmentation. Ablation studies confirm that the fuzzy enhancement branches, cross-representation attention, and joint multi-task learning each contribute to the overall performance. Statistical and qualitative analyses further demonstrate the stability of the results and the model’s ability to localize relevant lesion regions. The proposed framework provides an effective and interpretable approach to unified medical image classification and segmentation while maintaining a reasonable balance between predictive performance and computational cost.

## 1. Introduction

Currently, artificial intelligence and deep learning models are widely applied across various fields, including ecology, medicine, agriculture, environmental monitoring, intelligent transportation, and industrial automation [1,2,3,4,5]. In ecology, AI-based systems are used for wildlife monitoring, species identification, habitat analysis, and environmental change detection. In medicine, deep learning supports disease classification, organ and lesion segmentation, medical image enhancement, and computer-aided diagnosis. Despite the differences among these application domains, many tasks share common challenges, such as noisy data, low-contrast images, limited annotations, class imbalance, and variations in acquisition conditions [6,7,8,9,10,11,12,13]. Deep learning-based medical image analysis has demonstrated great capabilities in illness diagnosis, tissue characterization, and lesion segmentation with the quick development of artificial intelligence. Medical images frequently include low contrast, noise, imprecise lesion boundaries, intensity inhomogeneity, and substantial anatomical variability, which makes many clinical imaging issues challenging even with these advancements. These difficulties can make automatic systems less reliable, particularly when lesion location and disease detection are both necessary. Chest radiography and kidney image analysis are two representative yet difficult domains among clinically significant applications. Lesion segmentation shows the anatomical extent of aberrant findings, which is crucial for clinical interpretation, severity estimation, and follow-up evaluation, while image-level classification offers the diagnostic category. Analyzing kidney malignancies, renal cell carcinoma, and cystic renal masses in kidney imaging also necessitates precise lesion boundary delineation in addition to lesion classification. Because lesion size, form, and relationship to surrounding structures are directly related to diagnosis and therapy planning, this kind of delineation is crucial. Although current deep learning techniques have shown promise in either segmentation or classification, much research continues to handle these two tasks separately. The potential advantages of shared learning between local structural delineation and global semantic recognition are restricted by this division. Furthermore, most models now in use rely on a single image representation, which is often obtained from the original image or a traditional preprocessing phase. Nevertheless, a single representation might not adequately convey the variety of contrast and structural features required for reliable medical picture comprehension. Images with slight anomalies, blurry edges, or unclear intensity transitions exacerbate this problem.

As a result, picture augmentation is now a crucial stage in medical image analysis. While traditional techniques like filtering, adaptive contrast correction, and histogram equalization might enhance visual appearance, they frequently employ preset transformations and fail to properly reflect the uncertainty and slow intensity shifts that are typical in medical images. Because it can employ fuzzy membership functions to express confusing image structures and adjust enhancement power based on local image properties, fuzzy logic-based enhancement provides a more versatile option. However, rather than serving as complementary representations for more in-depth feature learning, improved images are typically just utilized as a preprocessing output. By allowing the network to concentrate on informative regions, channels, or features, attention mechanisms have also demonstrated high efficacy in medical picture analysis. To enhance classification and segmentation performance, recent research has employed spatial attention, channel attention, and self-attention. However, little attention has been dedicated to the problem of integrating numerous augmented visual representations by an explicit cross-representation attention mechanism. When various enhancement branches show distinct diagnostic cues that should be selectively merged rather than just concatenated, this approach is especially beneficial.

This paper suggests a Cross-Representation Attention-Based Neural Network with Fuzzy Image Enhancement for joint classification and segmentation of kidney and chest X-ray images to overcome these drawbacks. Using a novel fuzzy image improvement technique based on histogram spread, entropy, and standard deviation-driven local contrast changes, the suggested framework first creates three complementary enhanced representations from each input image. A Cross-Representation Attention Fusion module then processes these representations, learning how branches interact and combining their complementary data into a common feature space. A shared encoder and two task-specific heads then employ the fused features for simultaneous pixel-level segmentation and image-level classification.

The primary contributions of this work are summarized as follows:

A unified cross-representation attention-based neural network was developed for simultaneous medical image classification and segmentation.

A fuzzy image enhancement module was designed to generate three complementary representations from each input image using histogram spread, fuzzy entropy, and fuzzy standard deviation.

A Cross-Representation Attention Fusion (CRAF) mechanism was introduced to model multidirectional interactions among the three enhanced representations and adaptively integrate their complementary features.

The proposed framework was evaluated on chest X-ray and kidney image datasets, where both image-level diagnosis and lesion-level delineation are clinically relevant.

Quantitative comparison and ablation analysis were used to show performance improvements. The rest of the paper is structured as follows based on these motivations. Section 2 summarizes relevant research on attention-based fusion, segmentation, enhancement, and medical picture categorization. The suggested methodology, which includes fuzzy image improvement, Cross-Representation Attention Fusion, and the joint learning framework for kidney and chest image analysis, is explained in Section 3. The results and discussion of the experiment are presented in Section 4. The work is finally concluded and future research possibilities are outlined in Section 5.

## 2. Related Works

Artificial intelligence and deep learning are increasingly used in medical image analysis for automated classification, lesion localization, segmentation, image enhancement, and clinical decision support. Recent studies have moved beyond conventional convolutional networks toward attention mechanisms, multi-task learning, state-space models, and structure-preserving enhancement methods. However, challenges related to low contrast, ambiguous lesion boundaries, limited annotations, and heterogeneous image appearance remain important in chest X-ray and kidney image analysis.

### 2.1. Chest X-Ray Classification and Localization

Recent chest X-ray studies have investigated attention-guided learning to improve disease recognition. An et al. developed an attention-ensemble convolutional network for pneumonia detection and showed that combining complementary attention responses can improve classification robustness [14]. Kayumov et al. proposed an SE-attention-based ResNet50 architecture for COVID-19 detection from chest X-ray images [15]. Oltu et al. presented an automated attention-based framework for classifying chest radiographs and demonstrated the benefit of directing the network toward diagnostically relevant regions [16]. These studies indicate that attention can improve image-level classification by suppressing irrelevant background information. Recent research has also considered multi-label and multi-disease chest X-ray classification. Gopal et al. investigated multi-disease recognition using image-processing and deep-learning techniques [17]. A more comprehensive multi-label framework was subsequently developed to model dependencies among several thoracic findings instead of treating each abnormality independently [18]. DualAttNet introduced dual attention supervision for multi-label lesion detection and attempted to connect image-level prediction with lesion-related spatial information [19]. Nevertheless, these methods generally operate on one version of the input image and do not explicitly model interactions among multiple enhanced representations.

Classification alone provides limited information about the location and extent of abnormalities. Ou et al. therefore combined classification and semantic segmentation for tuberculosis lesions in chest radiographs using U-Net and attention-based variants [20]. Gopatoti et al. proposed dual-decoder attention networks for lung and infection segmentation, followed by classification of the extracted regions [21]. InfLocNet integrated a lightweight classification module with a U-Net++-based segmentation network to jointly detect disease and localize lung infection [22]. These studies support the clinical value of combining image-level diagnosis with lesion-level delineation, although their fusion strategies are mainly based on shared encoder features or decoder outputs rather than explicit cross-representation interaction.

### 2.2. Medical Image Enhancement

Medical images frequently contain low contrast, noise, reconstruction artifacts, and acquisition-dependent intensity variations. Yoo et al. reviewed recent deep-learning-based enhancement and reconstruction methods and emphasized their potential to improve image quality while reducing scan time or radiation exposure [23]. SSP-Net used a Siamese structure-preserving strategy to enhance medical images while reducing the risk of structural distortion [24]. Ye et al. developed a deep-learning enhancement method for cystoscopy images and reported improvements over conventional enhancement approaches [25]. These studies demonstrate that enhancement should preserve anatomical information rather than merely increase global contrast. Other recent studies have examined whether enhancement improves downstream interpretation. Steinmetz et al. showed that deep-learning-based contrast enhancement improved image quality and increased sensitivity for detecting vascular occlusions in poorly contrasted CT angiography [26]. Park et al. evaluated deep-learning image enhancement for glioma MRI across multiple institutions and reported improvements in signal-to-noise ratio, contrast-to-noise ratio, overall image quality, and lesion conspicuity [27]. These findings suggest that enhancement may support diagnostic analysis, but they also show that perceptual quality and downstream performance should be evaluated separately.

Deep perceptual enhancement methods have also been proposed to jointly correct contrast, luminance, and noise using learned residual representations [28]. In addition, fuzzy-driven enhancement has recently been used to generate complementary medical-image representations based on histogram spread, fuzzy entropy, and fuzzy standard deviation [29]. However, most existing methods produce a single enhanced image or evaluate several transformations independently. They do not explicitly learn how differently enhanced views should exchange complementary information during classification and segmentation.

### 2.3. Kidney and Renal Lesion Segmentation

Accurate segmentation of kidneys and renal lesions is essential for quantitative assessment, treatment planning, and follow-up. Ji et al. proposed ASD-Net, which combines asymmetric spatial and channel convolution to improve kidney and kidney-tumor segmentation [30]. Yao et al. developed an automated two-stage approach that first localized the kidney region and then segmented kidneys and tumors from CT images [31]. Hild et al. investigated automated segmentation of Wilms tumors and kidneys, demonstrating the feasibility of deep learning for pediatric renal-tumor delineation [32]. Recent methods have also emphasized small-lesion detection and multistage processing. Al-Battal et al. proposed a selective ensemble of U-Net-based models to improve liver and kidney lesion segmentation, particularly for small lesions [33]. Hao et al. developed a cascaded three-dimensional kidney segmentation framework for automated renal-tumor analysis [34]. These approaches improve spatial delineation but remain primarily segmentation-oriented and do not fully integrate image-level lesion classification into the same feature-learning process. Kidney-image analysis has also expanded toward classification and prognosis. Recent self-supervised learning has been applied to kidney-tumor classification from CT images [35], while end-to-end deep-learning systems have been developed for diagnosis of retroperitoneal neoplasms [36]. These studies confirm that deep networks can learn diagnostically relevant renal features, but classification and segmentation are still often treated as separate stages.

Attention-based segmentation methods attempt to emphasize relevant anatomical structures and suppress background responses. J-CaPA combined channel attention and pyramid attention in a Transformer-based U-Net for medical image segmentation [37]. More recent state-space architectures have sought to capture long-range dependencies with lower computational cost than conventional self-attention. Swin-UMamba adapted Mamba-based representation learning to medical image segmentation [38], while VMAXL-UNet combined Vision Mamba with lightweight extended LSTM units [39]. SliceMamba further introduced locally sensitive state-space modeling for efficient medical-image segmentation [40]. Other recent models have integrated Mamba with diffusion or multiscale feature learning. MambaDiff incorporated state-space modeling into a diffusion framework for three-dimensional medical-image segmentation [41]. Mamba-Sea used global-to-local state-space modeling to improve cross-domain generalization [42]. ADC-MambaNet focused on lightweight medical segmentation for resource-constrained environments [43]. Although these methods improve spatial modeling, they generally process a single image representation and do not explicitly define pairwise information transfer among multiple enhanced views. Multi-task learning provides another mechanism for combining complementary supervision. Lu et al. proposed MTL-OCA for simultaneous segmentation and classification of breast ultrasound images and used object-contextual attention to connect pixel-level and image-level information [44]. He et al. developed a multi-task framework for breast-tumor segmentation and classification using shared representations [45]. These studies demonstrate that classification and segmentation can reinforce each other when trained jointly. However, most multi-task architectures fuse tasks through a shared backbone rather than through explicit interactions among several differently enhanced input representations.

The recent literature reveals four main limitations. First, many chest X-ray methods focus on image-level classification, whereas lesion localization and segmentation remain less frequently integrated. Second, kidney-image methods achieve strong segmentation performance but often treat lesion classification as a separate task. Third, medical-image enhancement is commonly applied as an isolated preprocessing step and usually produces only one enhanced representation. Fourth, recent attention and state-space models mainly learn dependencies within a single representation or between two feature streams.

Therefore, limited research has investigated the explicit multidirectional fusion of three complementary enhanced representations within a unified classification–segmentation framework. The proposed method addresses this gap by generating histogram-, entropy-, and standard-deviation-based fuzzy representations, learning directed pairwise interactions through Cross-Representation Attention Fusion, and jointly optimizing image-level classification and lesion-level segmentation.

## 3. Materials and Methods

**Figure 1:**
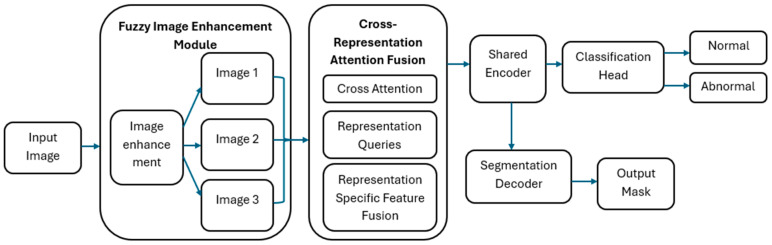
Overall framework of proposed methodology.

The overall framework of the proposed method is shown in Figure 1. Let the input medical image be denoted by *I*. The framework consists of three main stages: fuzzy image enhancement, cross-representation attention fusion, and joint classification and segmentation.

In the first stage, the input image is processed by the proposed fuzzy image enhancement module. Instead of generating a single enhanced image, the method produces three complementary enhanced representations, denoted by $I_{1}$, $I_{2}$, and $I_{3}$. These three images are obtained through different local contrast quantification strategies, namely histogram spread-based transformation, entropy-based transformation, and standard deviation-based transformation. The detailed explanation is provided in Algorithm 1. Each enhanced image emphasizes different structural and intensity characteristics of the original input.

**Algorithm 1.** Fuzzy Representation Image Enhancement**Input:** Medical image $I\left(x,y\right)$.**Output:** Three enhanced images $I_{h}$, $I_{e}$, and $I_{s}$.1. Normalize the input image to the range [0, 1].2. Calculate the dark, normal, and light fuzzy membership values:

$\mu_{D}\left(x,y\right),\;\mu_{N}\left(x,y\right),\;\mu_{L}\left(x,y\right).$

3. Refine each membership value using

$\mu_{F}^{r}\left(x,y\right)=\left\{\begin{array}{cc} 2{\left[\mu_{F}\left(x,y\right)\right]}^{2}, & 0\leq \mu_{F}\left(x,y\right)\leq 0.5,\\ 1-2{\left[1-\mu_{F}\left(x,y\right)\right]}^{2}, & 0.5<\mu_{F}\left(x,y\right)\leq 1. \end{array}\right.$

4. Calculate the local histogram spread and generate the first enhanced image:

$I_{1}^{enh}\left(x,y\right)=I{\left(x,y\right)}^{s\left(x,y\right)}.$

5. Calculate the local fuzzy entropy and generate the second enhanced image:

$I_{2}^{enh}\left(x,y\right)=I{\left(x,y\right)}^{a_{e}\left(x,y\right)}.$

6. Calculate the local fuzzy standard deviation and generate the third enhanced image:

$I_{3}^{enh}\left(x,y\right)=I{\left(x,y\right)}^{a_{s}\left(x,y\right)}.$

7. Defuzzify the three outputs:${\hat{I}}_{k}\left(x,y\right)=I_{k}^{enh}\left(x,y\right),\;k=1,2,3$.8. Assign the final enhanced images:

$I_{h}={\hat{I}}_{1},\;I_{e}={\hat{I}}_{2},\;I_{s}={\hat{I}}_{3}.$

**Return:** $I_{h}$, $I_{e}$, and $I_{s}$.

In the second stage, the three enhanced representations are passed to the Cross-Representation Attention Fusion module. This module is designed to learn the interdependence among multiple representations and to construct a fused feature space that preserves complementary discriminative information. Cross-attention is used to model the interaction among the representations, representation queries are generated to guide attention weighting, and representation-specific feature fusion is then performed to combine the resulting features into a unified embedding.

In the third stage, the fused feature representation is fed into a shared encoder. The shared encoder extracts deep semantic features that are beneficial for both image-level and pixel-level tasks. These encoded features are then passed to two task-specific branches: a classification head and a segmentation decoder. The classification head predicts whether the image belongs to the normal or abnormal category, or to a disease-specific class depending on the dataset setting. The segmentation decoder generates a lesion mask corresponding to the abnormal region. In this way, the network simultaneously learns disease recognition and lesion delineation.

Formally, the proposed framework can be expressed as:

(1) $$(I_{1},I_{2},I_{3})=E\left(I\right),$$

(2) $$F_{fused}=A\left(I_{1},I_{2},I_{3}\right),$$

(3) $$F_{enc}=S\left(F_{fused}\right),$$

(4) $$\hat{y}=C\left(F_{enc}\right),\hat{M}=D\left(F_{enc}\right),$$

where $E\left(\cdot \right)$ denotes the fuzzy enhancement module, $A\left(\cdot \right)$ denotes the Cross-Representation Attention Fusion module, $S\left(\cdot \right)$ denotes the shared encoder, $C\left(\cdot \right)$ denotes the classification head, and $D\left(\cdot \right)$ denotes the segmentation decoder. Here, $\hat{y}$ is the predicted class label and $\hat{M}$ is the predicted segmentation mask.

The main novelty of the proposed methodology lies in the coupling of multi-branch fuzzy enhancement and cross-representation attention learning within a unified multi-task framework. This design allows the network to exploit both enhanced visual quality and complementary feature interaction for more reliable medical image analysis.

### 3.1. Fuzzy Multi-Representation Image Enhancement

**Figure 2:**
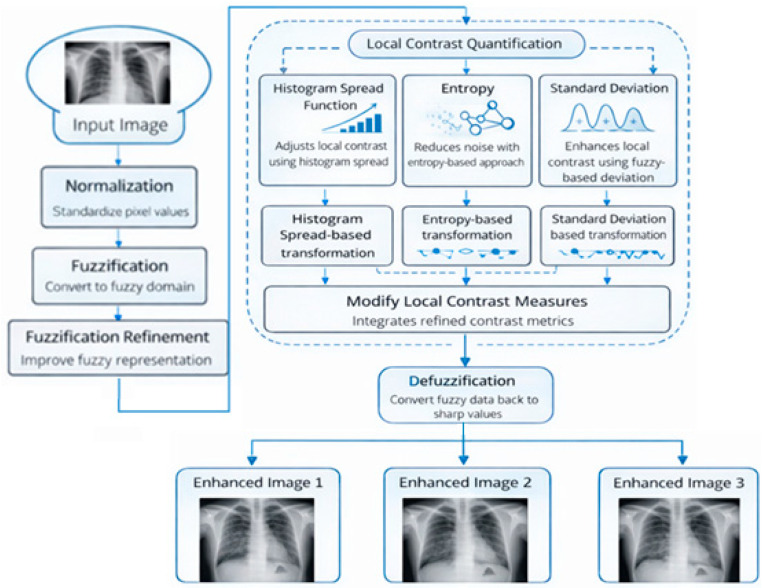
Image enhancement methodology.

The proposed image-enhancement method generates three complementary representations of each medical image. These representations are obtained using local histogram spread, fuzzy entropy, and fuzzy standard deviation. Each representation highlights different intensity and structural characteristics of the same image. The complete enhancement process consists of normalization, fuzzification, membership refinement, local contrast estimation, adaptive transformation, and defuzzification. The image enhancement methodology is illustrated in Figure 2.

#### 3.1.1. Image Normalization

Let $I\left(x,y\right)$ denote the intensity value of the input image at pixel position (*x*, *y*). First, the image is normalized to the interval [0, 1]:

(5) $$u\left(x,y\right)=\frac{I\left(x,y\right)-I_{\min}}{I_{\max}-I_{\min}+\varepsilon }.$$

here $I_{\min}$ is the minimum intensity value of the image, $I_{\max}$ is the maximum intensity value of the image, *ε* is a small positive constant preventing division by zero, $u\left(x,y\right)$ is the normalized pixel intensity. Consequently,

(6) $$0\leq u\left(x,y\right)\leq 1.$$

#### 3.1.2. Fuzzy Membership Functions

To obtain smooth transitions among dark, normal-intensity, and light image regions, symmetric double-sigmoid fuzzy membership functions are used instead of piecewise-linear functions. These functions avoid abrupt changes at fixed intensity thresholds and better represent the gradual intensity transitions commonly observed in medical images.

**Figure 3:**
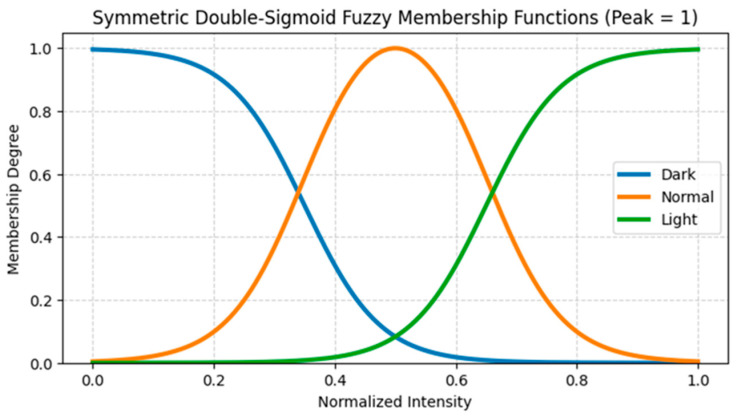
Symmetric double-sigmoid fuzzy membership functions for dark, normal-intensity, and light image regions. The horizontal axis represents normalized intensity, while the vertical axis represents the fuzzy membership degree.

Figure 3 illustrates the three membership functions over the normalized intensity interval [0, 1]. The dark membership is high for low-intensity pixels and gradually decreases as the intensity increases. The light membership behaves symmetrically and gradually increases toward high-intensity regions. The normal-intensity membership reaches its maximum near the center of the normalized intensity range.

Let $u\left(x,y\right)\in \left[0,\;1\right]$ denote the normalized intensity of the pixel located at (*x*, *y*). The preliminary dark membership function is defined as:

(7) $$m_{D}\left(x,y\right)=\frac{1}{1+\exp\left[\alpha \left(u\left(x,y\right)-c_{D}\right)\right]}.$$

The preliminary light membership function is defined symmetrically as

(8) $$m_{L}\left(x,y\right)=\frac{1}{1+\exp\left[-\alpha \left(u\left(x,y\right)-c_{L}\right)\right]}.$$

Here, α>0 controls the steepness of the sigmoid functions. The parameter $c_{D}$ is the transition center between the dark and normal-intensity regions, whereas $c_{L}$ is the transition center between the normal-intensity and light regions, with $c_{D}<c_{L}$.

In the present implementation, the transition centers are selected symmetrically around the midpoint of the normalized intensity range:

(9) $$c_{D}=0.35,\;c_{L}=0.65.$$

The midpoint of the normal-intensity region is therefore

(10) $$c_{N}=\frac{c_{D}+c_{L}}{2}=0.5.$$

The preliminary normal-intensity membership is constructed as the product of an increasing sigmoid and a decreasing sigmoid:

(11) $$m_{N}\left(x,y\right)=\frac{1}{\kappa }\left[\frac{1}{1+\exp\left[-\alpha \left(u\left(x,y\right)-c_{D}\right)\right]}\right]\left[\frac{1}{1+\exp\left[\alpha \left(u\left(x,y\right)-c_{L}\right)\right]}\right].$$

The normalization factor *κ* is selected so that the preliminary normal-intensity membership reaches a maximum value of 1 at $u\left(x,y\right)=c_{N}$:

(12) $$\kappa =\left[\frac{1}{1+\exp\left[-\alpha \left(c_{N}-c_{D}\right)\right]}\right]\left[\frac{1}{1+\exp\left[\alpha \left(c_{N}-c_{L}\right)\right]}\right].$$

Accordingly,

(13) $$m_{N}\left(c_{N}\right)=1.$$

The preliminary membership functions $m_{D}\left(x,y\right)$, $m_{N}\left(x,y\right)$, and $m_{L}\left(x,y\right)$ describe smooth fuzzy transitions. However, their sum is not necessarily equal to one. Therefore, they are normalized as follows:

(14) $$\mu_{F}\left(x,y\right)=\frac{m_{F}\left(x,y\right)}{m_{D}\left(x,y\right)+m_{N}\left(x,y\right)+m_{L}\left(x,y\right)},\;F\in \left\{D,N,L\right\}.$$

More explicitly, the normalized dark membership is:

(15) $$\mu_{D}\left(x,y\right)=\frac{m_{D}\left(x,y\right)}{m_{D}\left(x,y\right)+m_{N}\left(x,y\right)+m_{L}\left(x,y\right)}.$$

The normalized normal-intensity membership is:

(16) $$\mu_{N}\left(x,y\right)=\frac{m_{N}\left(x,y\right)}{m_{D}\left(x,y\right)+m_{N}\left(x,y\right)+m_{L}\left(x,y\right)}.$$

The normalized light membership is:

(17) $$\mu_{L}\left(x,y\right)=\frac{m_{L}\left(x,y\right)}{m_{D}\left(x,y\right)+m_{N}\left(x,y\right)+m_{L}\left(x,y\right)}.$$

After normalization, the fuzzy memberships satisfy:

(18) $$\mu_{D}\left(x,y\right)+\mu_{N}\left(x,y\right)+\mu_{L}\left(x,y\right)=1.$$

This normalization guarantees that every pixel is represented by a valid fuzzy partition. For low normalized intensity values, that is $u\left(x,y\right)\to 0$, the dark membership becomes dominant:

(19) $$\mu_{D}\left(x,y\right)>\mu_{N}\left(x,y\right),\mu_{D}\left(x,y\right)>\mu_{L}\left(x,y\right).$$

At the central normalized intensity, $u\left(x,y\right)=c_{N}=0.5$, the normal-intensity membership becomes dominant:

(20) $$\mu_{N}\left(x,y\right)>\mu_{D}\left(x,y\right),\mu_{N}\left(x,y\right)>\mu_{L}\left(x,y\right).$$

For high normalized intensity values, that is $u\left(x,y\right)\to 1$, the light membership becomes dominant:

(21) $$\mu_{L}\left(x,y\right)>\mu_{D}\left(x,y\right),\mu_{L}\left(x,y\right)>\mu_{N}\left(x,y\right).$$

The steepness parameter *α* controls the degree of overlap among the three membership functions. A smaller value of *α* produces smoother and wider transitions, whereas a larger value produces sharper separation among the fuzzy classes. In the experiments, *α* was selected using the validation dataset.

#### 3.1.3. Fuzzification Refinement

After the initial fuzzification stage, the membership values are refined to increase the distinction between weak and strong memberships. The refined fuzzy membership is defined as:

(22) $$\mu_{F}^{r}\left(x,y\right)=\left\{\begin{array}{cc} 2{\left[\mu_{F}\left(x,y\right)\right]}^{2}, & 0\leq \mu_{F}\left(x,y\right)\leq \frac{1}{2},\\ 1-2{\left[1-\mu_{F}\left(x,y\right)\right]}^{2}, & \frac{1}{2}<\mu_{F}\left(x,y\right)\leq 1. \end{array}\right.$$

Here:

- $\mu_{F}\left(x,y\right)$ is the original fuzzy membership value of pixel (*x*, *y*);
- $\mu_{F}^{r}\left(x,y\right)$ is the refined fuzzy membership value;
- $F\in \left\{D,N,L\right\}$ denotes the dark, normal-intensity, or light fuzzy set.

For membership values lower than or equal to 0.5, the transformation reduces weak memberships:

$$\mu_{F}^{r}\left(x,y\right)=2{\left[\mu_{F}\left(x,y\right)\right]}^{2}.$$

For membership values greater than 0.5, the transformation strengthens dominant memberships:

$$\mu_{F}^{r}\left(x,y\right)=1-2{\left[1-\mu_{F}\left(x,y\right)\right]}^{2}.$$

The transformation preserves the interval

$$0\leq \mu_{F}^{r}\left(x,y\right)\leq 1,$$

and satisfies the following characteristic points:

$$\mu_{F}^{r}=0\;\mathrm{when}\;\mu_{F}=0,$$

$$\mu_{F}^{r}=0.5\;\mathrm{when}\;\mu_{F}=0.5,$$

and

$$\mu_{F}^{r}=1\;\mathrm{when}\;\mu_{F}=1.$$

Thus, the refinement operation suppresses uncertain low memberships and reinforces high memberships, thereby improving the separation among dark, normal-intensity, and light image regions.

#### 3.1.4. Local Histogram and Cumulative Distribution

At this stage, the local contrast is estimated from the cumulative distribution of gray-level intensities. Let $h\left(j\right)$ denote the local histogram of the image. The corresponding cumulative distribution function is defined as:

(23) $$CDF\left(i\right)=\underset{j\leq i}{\sum }h\left(j\right).$$

Using the cumulative distribution, the local histogram spread is calculated as

(24) $$h_{F}\left(x,y\right)=\max\left(CDF\right)-\min\left(CDF\right).$$

Here, $h_{F}\left(x,y\right)$ characterizes the local spread of intensity values around pixel (*x*, *y*). A larger value of $h_{F}\left(x,y\right)$ indicates a wider intensity distribution and, consequently, a higher local contrast.

#### 3.1.5. Histogram-Spread-Based Enhancement

Based on the local histogram spread, the adaptive contrast-control parameter is defined as:

(25) $$s\left(x,y\right)={\left(h_{F}\left(x,y\right)\right)}^{a},a>0.$$

Here, *a* is the parameter controlling the contrast enhancement. In the present study, the value *a* = 0.7 is used. The first enhanced image, obtained from the histogram-spread-based local contrast modification, is then expressed as:

(26) $$I_{1}^{enh}\left(x,y\right)=I{\left(x,y\right)}^{\;s\left(x,y\right)}.$$

Thus, the local contrast is adaptively adjusted according to the spread of the cumulative histogram. Regions with different local intensity distributions receive different enhancement strengths.

#### 3.1.6. Fuzzy-Entropy-Based Enhancement

In the second branch, local contrast is modified according to fuzzy entropy. The local fuzzy entropy is defined as

(27) $$\varepsilon \left(\mu_{F}\right)=-\frac{\sum_{i=1}^{nm}\left[\mu_{F}^{r}\left(f_{i}\right)\ln\mu_{F}^{r}\left(f_{i}\right)+\left(1-\mu_{F}^{r}\left(f_{i}\right)\right)\ln\left(1-\mu_{F}^{r}\left(f_{i}\right)\right)\right]}{\log\left(nm\right)}.$$

Here $\varepsilon \left(\mu_{F}\right)$ is the local fuzzy entropy, $\mu_{F}^{r}\left(f_{i}\right)$ is the refined fuzzy membership value, *nm* denotes the number of samples in the local region. Using the local fuzzy entropy, the adaptive enhancement exponent is determined as:

(28) $$a\left(x,y\right)=a_{\min}+\left(a_{\max}-a_{\min}\right){\left(\varepsilon \left(\mu_{F}\right)\right)}^{s}.$$

In this work, the parameters are set as $s=1.3,\;a_{\min}=0.6,\;a_{\max}=1.4$. The second enhanced image is therefore obtained as:

(29) $$I_{2}^{enh}\left(x,y\right)=I{\left(x,y\right)}^{\;a\left(x,y\right)}.$$

This branch adjusts the enhancement strength according to the amount of local fuzzy information. Regions with different entropy levels are enhanced differently.

#### 3.1.7. Fuzzy-Standard-Deviation-Based Enhancement

In the third branch, the enhancement is controlled by fuzzy standard deviation. First, the mean image intensity is computed as:

(30) $$ML=\frac{1}{HW}\overset{H}{\underset{x=1}{\sum }}\overset{W}{\underset{y=1}{\sum }}I\left(x,y\right),$$

where *H* and *W* denote the height and width of the image, respectively. The quantity *ML* represents the average intensity level of the image. Next, the fuzzy standard deviation is calculated as:

(31) $$\sigma_{F}\left(x,y\right)=\sqrt{\frac{\sum_{\left(i,j)\in \Omega (x,y\right)}{\left(I\left(i,j\right)-ML\right)}^{2}\mu_{F}^{r}\left(i,j\right)}{\sum_{\left(i,j)\in \Omega (x,y\right)}\mu_{F}^{r}\left(i,j\right)}}.$$

Here $\Omega \left(x,y\right)$ denotes the local neighborhood centered at (*x*, *y*), $\mu_{F}^{r}\left(i,j\right)$ is the refined fuzzy membership value at pixel (*i*, *j*). Using the fuzzy standard deviation, the adaptive exponent is defined as:

(32) $$a\left(x,y\right)=a_{\max}-\left(a_{\max}-a_{\min}\right)\sigma_{F}{\left(x,y\right)}^{s}.$$

In this branch, the parameters are chosen as $s=1.3,\;a_{\min}=0.8,\;a_{\max}=1.2$. The third enhanced image is then obtained as:

(33) $$I_{3}^{enh}\left(x,y\right)=I{\left(x,y\right)}^{\;a\left(x,y\right)}.$$

This formulation allows the contrast enhancement to be adapted according to the fuzzy local intensity dispersion. Regions with low fuzzy standard deviation are enhanced more strongly, whereas high-variation regions are modified more conservatively.

#### 3.1.8. Defuzzification and Final Enhanced Images

At the final stage, the enhanced fuzzy-domain outputs are mapped back to the image domain through defuzzification. The defuzzified output images are defined as

(34) $${\hat{I}}_{k}\left(x,y\right)=I_{k}^{enh}\left(x,y\right),\;k=1,2,3.$$

Here ${\hat{I}}_{k}\left(x,y\right)$ is the defuzzified intensity value at pixel (*x*, *y*), $I_{k}^{enh}\left(x,y\right)$ is the enhanced output generated by the *k*-th branch, *k* = 1 denotes histogram-spread-based enhancement, *k* = 2 denotes fuzzy-entropy-based enhancement, *k* = 3 denotes fuzzy-standard-deviation-based enhancement. Accordingly, the three final enhanced images are expressed as:

(35) $${\hat{I}}_{1}\left(x,y\right)=I_{1}^{enh}\left(x,y\right),$$

(36) $${\hat{I}}_{2}\left(x,y\right)=I_{2}^{enh}\left(x,y\right),$$

and

(37) $${\hat{I}}_{3}\left(x,y\right)=I_{3}^{enh}\left(x,y\right).$$

For subsequent processing, these three outputs are denoted as

(38) $$I_{h}={\hat{I}}_{1},\;I_{e}={\hat{I}}_{2},\;I_{s}={\hat{I}}_{3},$$

where $I_{h}$, $I_{e}$, and $I_{s}$ represent the histogram-spread-enhanced, fuzzy-entropy-enhanced, and fuzzy-standard-deviation-enhanced images, respectively.

**Figure 4:**
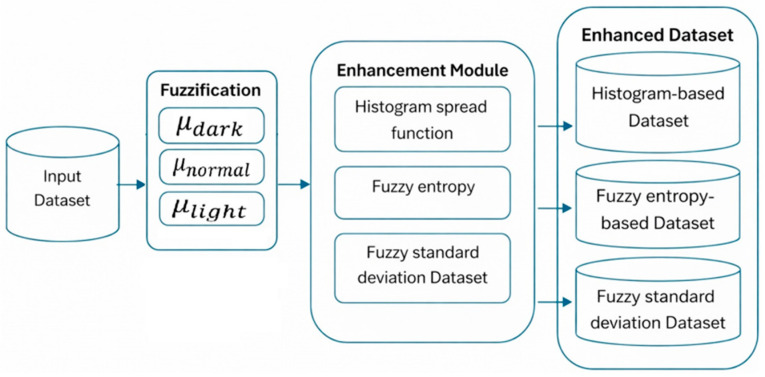
Data augmentation by image enhancement.

In addition to image-level enhancement, the same procedure can also be applied dataset-wise to construct an enhanced dataset, as illustrated in Figure 4.

In this setting, each original image is transformed into three enhanced versions, enabling the network to learn from multiple representation-specific datasets.

### 3.2. Cross-Representation Attention Fusion

**Figure 5:**
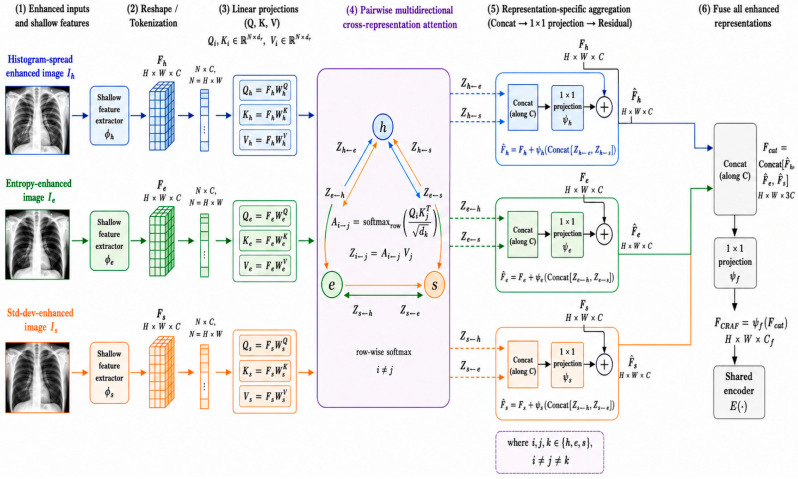
Cross-Representation Attention Fusion Module.

After fuzzy enhancement, the proposed framework receives three complementary images: the histogram-spread-enhanced image $I_{h}$, the fuzzy-entropy-enhanced image $I_{e}$, and the fuzzy-standard-deviation-enhanced image $I_{s}$. Although these images are generated from the same original input, they emphasize different contrast and structural properties. The purpose of the Cross-Representation Attention Fusion module is to explicitly model the relationships among the three representations and integrate their complementary information into a unified feature map (see Figure 5).

The three enhanced images are first processed by shallow feature-extraction branches:

(39) $$F_{h}=\phi_{h}\left(I_{h}\right),\;F_{e}=\phi_{e}\left(I_{e}\right),\;F_{s}=\phi_{s}\left(I_{s}\right),$$

where $\phi_{h}$, $\phi_{e}$, and $\phi_{s}$ denote the shallow feature extractors for the histogram, entropy, and standard-deviation branches, respectively.

Each extracted feature map has dimensions

(40) $$F_{i}\in R^{H\times W\times C},i\in \left\{h,e,s\right\},$$

where *H* and *W* are the spatial dimensions and *C* is the number of feature channels.

Before calculating attention, each feature map is reshaped into a sequence of spatial tokens:

(41) $$F_{i}\in R^{N\times C},\;N=H\times W.$$

Here, *N* denotes the number of spatial positions.

#### 3.2.1. Distinction from Conventional Attention Mechanisms

The proposed CRAF module differs from averaging, concatenation, self-attention, and conventional cross-attention as shown in Table 1. Simple averaging treats all three enhanced representations equally and does not identify which branch contains more useful information at a particular spatial location. Concatenation retains all branch features but does not explicitly model the relationships among them. Instead, the subsequent convolution layers must learn the interactions indirectly. Self-attention calculates dependencies within a single representation. Therefore, it can model spatial relationships inside one enhanced image but cannot directly transfer information among the histogram, entropy, and standard-deviation-enhanced branches. Conventional cross-attention usually involves two inputs. One representation produces the queries, while another provides the keys and values. The interaction is commonly one-directional unless a second reverse-attention operation is added. In contrast, the proposed CRAF module performs directed pairwise attention among all three enhanced representations. Each representation acts both as a target branch and as an information source. This produces six directed interactions and enables multidirectional transfer of complementary features.

The interaction structure of CRAF is symmetric because every branch communicates with the other two branches. However, the individual attention operations are directional. Therefore, attention from branch *j* to branch *i* is generally different from attention from branch *i* to branch *j*.

#### 3.2.2. Query, Key, and Value Generation

For each representation $F_{i}$, the query, key, and value matrices are generated using learnable linear projections:

(42) $$Q_{i}=F_{i}W_{i}^{Q},K_{i}=F_{i}W_{i}^{K},V_{i}=F_{i}W_{i}^{V},$$

where $W_{i}^{Q}$, $W_{i}^{K}$, and $W_{i}^{V}$ are trainable projection matrices for branch *i*.

Separate projection parameters are used for the histogram, entropy, and standard-deviation branches because the three representations contain different intensity and structural characteristics. The projected tensors have the following dimensions:

(43) $$Q_{i},K_{i}\in R^{N\times d_{k}},V_{i}\in R^{N\times d_{v}},$$

where $d_{k}$ is the query and key embedding dimension and $d_{v}$ is the value embedding dimension.

The term $d_{k}$ is not the original channel dimension *C*. It is the dimensionality of the projected query and key vectors. The scaling factor $\sqrt{d_{k}}$ is used to prevent excessively large dot-product values and stabilize gradient-based optimization.

#### 3.2.3. Pairwise Cross-Representation Attention

For two different representations *i* and *j*, the directed attention from source branch *j* to target branch *i* is calculated as:

(44) $$A_{i\leftarrow j}={\mathrm{softmax}}_{\mathrm{row}}\left(\frac{Q_{i}K_{j}^{T}}{\sqrt{d_{k}}}\right),\;i\neq j.$$

Here, $Q_{i}$ is generated from the target representation, while $K_{j}$ is generated from the source representation.

The attention matrix has dimensions

(45) $$A_{i\leftarrow j}\in R^{N\times N}.$$

The softmax function is applied row-wise. Consequently, each target query position assigns normalized weights to all source positions:

(46) $$\sum_{n=1}^{N}A_{i\leftarrow j}\left(m,n\right)=1$$

for every target position *m*.

The feature response transferred from representation *j* to representation *i* is

(47) $$Z_{i\leftarrow j}=A_{i\leftarrow j}V_{j}.$$

The proposed three-branch design produces six directed pairwise responses:

(48) $$Z_{h\leftarrow e},Z_{h\leftarrow s},$$

(49) $$Z_{e\leftarrow h},Z_{e\leftarrow s},$$

and

(50) $$Z_{s\leftarrow h},Z_{s\leftarrow e}.$$

Although every pair of branches communicates in both directions, the responses are generally not identical:

(51) $$A_{i\leftarrow j}\neq A_{j\leftarrow i}.$$

This is because the target queries, source keys, and branch-specific projection parameters differ.

#### 3.2.4. Representation-Specific Aggregation

Each target branch receives two complementary attention responses from the other branches. These responses are concatenated and projected using a 1 × 1 convolution:

(52) $$R_{i}=\psi_{i}\left(Concat\left[Z_{i\leftarrow j},Z_{i\leftarrow k}\right]\right),$$

where $i,j,k\in \left\{h,e,s\right\}$, i≠j, i≠k, and j≠k. The function $\psi_{i}\;$ denotes a branch-specific 1×1 convolution followed by normalization and nonlinear activation. A residual connection is then applied:

(53) $${\hat{F}}_{i}=F_{i}+R_{i}.$$

More explicitly,

(54) $${\hat{F}}_{h}=F_{h}+\psi_{h}\left(Concat\left[Z_{h\leftarrow e},Z_{h\leftarrow s}\right]\right),$$

(55) $${\hat{F}}_{e}=F_{e}+\psi_{e}\left(Concat\left[Z_{e\leftarrow h},Z_{e\leftarrow s}\right]\right),$$

and

(56) $${\hat{F}}_{s}=F_{s}+\psi_{s}\left(Concat\left[Z_{s\leftarrow h},Z_{s\leftarrow e}\right]\right).$$

The residual connection preserves the original representation-specific information while adding complementary information obtained from the other two branches.

#### 3.2.5. Final Cross-Representation Fusion

The three refined branch features are concatenated along the channel dimension:

(57) $$F_{\mathrm{cat}}=\mathrm{Concat}\left[{\hat{F}}_{h},{\hat{F}}_{e},{\hat{F}}_{s}\right].$$

The final fused representation is calculated as:

(58) $$F_{\mathrm{CRAF}}=\psi_{f}\left(F_{\mathrm{cat}}\right),$$

where $\psi_{f}$ denotes the final learnable projection implemented using a 1×1 convolution, normalization, and nonlinear activation.

The resulting feature map $F_{\mathrm{CRAF}}$ is then supplied to the shared encoder:

(59) $$F_{\mathrm{enc}}=E\left(F_{\mathrm{CRAF}}\right),$$

where $E\left(\cdot \right)$ denotes the shared encoder. The proposed fusion process can therefore be summarized as:

(60) $$\left\{F_{h},F_{e},F_{s}\right\}\to \mathrm{pairwise}\;\mathrm{directed}\;\mathrm{attention}\to \left\{{\hat{F}}_{h},{\hat{F}}_{e},{\hat{F}}_{s}\right\}\to F_{\mathrm{CRAF}}.$$

#### 3.2.6. Advantages and Computational Considerations

The main advantage of CRAF is that it performs explicit, adaptive, and spatially selective information transfer among all three enhancement branches. Unlike averaging, it does not assign equal importance to every representation. Unlike concatenation, it does not rely solely on later convolutional layers to discover relationships among the branches. Unlike self-attention, it directly exchanges information across different enhanced representations. Unlike standard two-stream cross-attention, it supports three representations and multidirectional pairwise interactions.

The module introduces additional computational cost because six directed attention operations are required. To control this overhead, attention is applied after shallow feature extraction and uses reduced query and key dimensions $d_{k}<C$. The computational benefit of the proposed module is evaluated experimentally by comparing CRAF with averaging, concatenation, self-attention, and conventional cross-attention using classification performance, segmentation performance, parameter count, FLOPs, memory consumption, and inference time.

### 3.3. Detailed Neural Network Architecture

The proposed network uses a custom U-Net-like multi-task architecture rather than a standard ResNet or an unchanged U-Net. The framework consists of three shallow feature-extraction branches, the proposed CRAF module, a four-stage shared convolutional encoder, a classification head, and a segmentation decoder with encoder–decoder skip connections. The same backbone configuration is used for the chest X-ray and kidney experiments. The only dataset-specific components are the output dimensions of the classification and segmentation heads. All tensor dimensions reported below assume a batch size of *B* and an input resolution of 512×512. Each fuzzy-enhanced image is represented as:

$$I_{i}\in R^{B\times 1\times 512\times 512},i\in \left\{h,e,s\right\},$$

where $I_{h}$, $I_{e}$, and $I_{s}$ denote the histogram, entropy, and standard-deviation-enhanced images, respectively.

#### 3.3.1. Shallow Feature-Extraction Branches

Each enhanced image is processed by an independent shallow feature-extraction branch. The three branches have identical layer configurations but do not share convolutional parameters because each enhanced representation emphasizes different image characteristics.

Each branch contains two convolutional blocks. A convolutional block consists of convolution, batch normalization, and ReLU activation:

$$\mathrm{ConvBlock}\left(X\right)=\mathrm{ReLU}\left(\mathrm{BN}\left(\mathrm{Conv}\left(X\right)\right)\right).$$

The branch output is

$$F_{i}^{\left(0\right)}=\phi_{i}\left(I_{i}\right)\in R^{B\times 32\times 128\times 128}.$$

Spatial reduction before attention limits the quadratic memory cost of dot-product attention while retaining the principal lesion structures.

#### 3.3.2. CRAF Input and Attention Projections

Before pairwise attention calculation, each branch feature is adaptively pooled to 32×32:

$$F_{i}^{a}={\mathrm{AAP}}_{32\times 32}\left(F_{i}^{\left(0\right)}\right)\in R^{B\times 32\times 32\times 32}.$$

The pooled feature is flattened into N=32×32=1024 spatial tokens:

$${\bar{F}}_{i}\in R^{B\times 1024\times 32}.$$

The branch-specific query, key, and value projections are

$$Q_{i}={\bar{F}}_{i}W_{i}^{Q},\;K_{i}={\bar{F}}_{i}W_{i}^{K},\;V_{i}={\bar{F}}_{i}W_{i}^{V},$$

where

$$Q_{i},K_{i},V_{i}\in R^{B\times 1024\times 64}.$$

Thus, the attention embedding dimensions are $d_{k}=d_{v}=64$. Six directed pairwise interactions are calculated:

h←e, h←s, e←h, e←s, s←h, s←e.

For each target branch, the two incoming responses are concatenated, projected from 128 to 32 channels, restored to 128×128, and combined with the original shallow feature through a residual connection. The three refined branch outputs are concatenated:

$$F_{\mathrm{cat}}=\mathrm{Concat}\left[{\hat{F}}_{h},{\hat{F}}_{e},{\hat{F}}_{s}\right]\in R^{B\times 96\times 128\times 128}.$$

A 1×1  convolution projects the concatenated tensor to 64 channels:

$$F_{\mathrm{CRAF}}\in R^{B\times 64\times 128\times 128}.$$

#### 3.3.3. Shared Encoder

The shared encoder contains four resolution stages and one bottleneck stage. Each encoder stage contains two 3×3 convolutional layers, each followed by batch normalization and ReLU activation. Spatial downsampling is performed using 2×2 max pooling.

The encoder generates the skip features $S_{1},\;S_{2},\;S_{3},\;S_{4},\;$ which are supplied to the corresponding decoder stages.

#### 3.3.4. Classification Head

The classification head operates on the bottleneck feature map. Global average pooling converts the spatial feature tensor into a feature vector:

$$g=\mathrm{GAP}\left(F_{B}\right)\in R^{B\times 1024}.$$

The classification head is defined as:

$$g_{1}=\mathrm{Dropout}\left(\mathrm{ReLU}\left(W_{1}g+b_{1}\right)\right),$$

where $g_{1}\in R^{B\times 512}$, followed by

$${\hat{y}}_{\mathrm{cls}}=W_{2}g_{1}+b_{2}.$$

The final layer contains $C_{\mathrm{cls}}$ output units, where $C_{\mathrm{cls}}$ is the number of image-level classes in the corresponding dataset.

#### 3.3.5. Segmentation Decoder

The segmentation decoder contains four upsampling stages. Each stage uses a 2×2 transposed convolution, concatenation with the corresponding encoder skip feature, and two 3×3 convolutional blocks.

The decoder progressively restores the feature resolution from 8×8 to 128×128. Two additional bilinear upsampling operations restore the segmentation map to the original 512×512 resolution.

The final segmentation logits are produced using a 1×1 convolution:

$${\hat{Y}}_{\mathrm{seg}}\in R^{B\times C_{\mathrm{seg}}\times 512\times 512},$$

where $C_{\mathrm{seg}}$ denotes the number of segmentation output channels.

For binary segmentation, $C_{\mathrm{seg}}=1$, and a sigmoid activation is used. For multi-class segmentation, $C_{\mathrm{seg}}$ equals the number of segmentation classes, and softmax is applied along the channel dimension. Detailed architecture of the proposed network is presented in Appendix A, Table A1.

The architecture was kept identical across the chest X-ray and kidney experiments to ensure that differences in performance were not caused by dataset-specific backbone modifications. The shallow feature branches, CRAF module, shared encoder, decoder stages, skip connections, and classification head structure remained unchanged. Only the dimensions of the final classification and segmentation layers were adapted to the number of target classes in each dataset. The use of encoder–decoder skip connections enables the decoder to recover fine spatial information that may be lost during downsampling. The deeper encoder features provide high-level semantic information, while the shallower skip features retain lesion boundaries and local anatomical details. The classification head uses the bottleneck representation because it contains the most abstract and globally discriminative features.

### 3.4. Datasets, Annotations, and Data Partitioning

The proposed framework was evaluated on two independently constructed medical image collections: a chest X-ray dataset and a kidney CT dataset as shown in Table 2. The chest X-ray dataset was assembled from four publicly available sources to support classification and segmentation of normal cases and four thoracic abnormalities. The kidney dataset was derived from the two-dimensional version of the KiTS23 dataset and was used to identify normal kidney tissue, solid renal tumors, including renal cell carcinoma, and cystic renal masses. The two datasets were processed and evaluated separately because they represent different imaging modalities, anatomical regions, label structures, and clinical tasks. Consequently, an independent model instance was trained for each dataset using the same proposed architecture but separately optimized parameters.

#### 3.4.1. Chest X-Ray Dataset

The chest X-ray collection contained 41,467 images obtained from CheXpert, the RSNA Pneumonia Detection dataset, the SIIM–ACR Pneumothorax Segmentation dataset, CheXpert-seg, and the Lung Opacity subset of the COVID-19 Radiography Database. All images were converted or resized to a uniform spatial resolution of (512 × 512) pixels.

The normal class consisted of 22,381 images obtained from CheXpert. Because these images did not contain a target abnormality, their corresponding segmentation targets were represented by empty binary masks in which every pixel was assigned a value of 0.

The pneumonia class contained 9555 images obtained from the RSNA Pneumonia Detection dataset. The associated binary masks represented pulmonary regions affected by consolidation or other pneumonia-related opacity patterns. The pneumothorax class contained 2669 images obtained from the SIIM–ACR Pneumothorax Segmentation dataset, with pixel-level binary masks delineating pleural air collections. The pleural-effusion class contained 850 images from CheXpert-seg, for which the abnormal fluid-accumulation regions were represented by manually delineated polygon-based masks. The lung-opacity class contained 6012 images obtained from the Lung Opacity subset of the COVID-19 Radiography Database, with binary masks indicating opacified pulmonary regions.

The resulting chest X-ray collection was partitioned into 33,174 training images, 4146 validation images, and 4147 test images. The approximate partition ratio was 80:10:10. Partitioning was performed separately within each class to preserve the class distribution across the three subsets. All image enhancement and training-time augmentation operations were performed after data partitioning. The three enhanced representations generated from an original image were retained in the same subset as the source image, thereby preventing an original image and its enhanced copies from appearing in different data partitions.

#### 3.4.2. Kidney Dataset

The kidney experiments were conducted using a two-dimensional image collection derived from KiTS23, hereafter referred to as KiTS23-2D. The collection contained 108,293 axial CT images, each represented at a spatial resolution of 512 × 512 pixels. The segmentation annotations were encoded as categorical masks identifying healthy kidney tissue, solid renal tumor tissue, and cystic renal structures.

The normal kidney class contained 63,179 images. In the corresponding categorical masks, healthy kidney tissue was assigned a pixel value of 1. The solid-tumor class contained 31,142 images showing renal tumors, including renal cell carcinoma regions, which were assigned a mask value of 2. The cystic renal mass class contained 13,972 images containing fluid-filled cystic structures, which were assigned a mask value of 3.

The KiTS23-2D collection was divided into 86,633 training images, 10,829 validation images, and 10,831 test images, corresponding approximately to an 80:10:10 split. As in the chest X-ray experiments, dataset partitioning was completed before fuzzy enhancement and conventional data augmentation. All enhanced representations generated from a given CT image remained in the same partition as the original image.

#### 3.4.3. Annotation Processing

For both datasets, the segmentation targets were converted into pixel-aligned masks with a spatial resolution of 512 × 512 pixels. For normal chest radiographs, an empty mask was used because no target lesion was present. For abnormal chest radiographs, the available binary or polygon-based annotations were converted into binary masks, where a value of 1 indicated the target pathological region and a value of 0 indicated the background. For the kidney dataset, the original categorical annotations were retained to distinguish healthy kidney tissue, solid renal tumor tissue, and cystic tissue. During binary task-specific evaluation, the relevant target category was separated from the background as required by the segmentation task. Image resizing and geometric preprocessing were applied identically to each image and its corresponding mask to maintain pixel-wise alignment. No enhanced or augmented image was treated as an independent patient or independently partitioned sample. Enhanced variants were generated only after the source images had been assigned to the training, validation, or test subset. This procedure reduced the possibility of direct image-level leakage between the subsets.

### 3.5. Loss Function

The proposed Cross-Representation Attention-based Neural Network is trained in a multi-task manner to perform both image-level classification and pixel-level segmentation. Since these two tasks are complementary but different in objective, the total loss function is defined as a weighted combination of classification loss and segmentation loss.

Let $\hat{y}$ denote the predicted class probability vector and *y* denote the ground-truth class label. The classification branch is optimized using categorical cross-entropy loss:

(61) $$L_{cls}=-\sum_{c=1}^{C}y_{c}\log\left({\hat{y}}_{c}\right),$$

where *C* is the number of classes.

For the segmentation branch, let $\hat{M}$ and *M* denote the predicted and ground-truth masks, respectively. To encourage accurate overlap between the predicted lesion region and the true annotated lesion region, Dice loss is employed:

(62) $$L_{Dice}=1-\frac{2\sum \left(\hat{M}\cdot M\right)+\epsilon }{\sum \hat{M}+\sum M+\epsilon },$$

where *ϵ* is a small constant to avoid numerical instability.

In addition, binary cross-entropy loss can be incorporated into the segmentation branch to improve pixel-wise discrimination:

(63) $$L_{BCE}=-\frac{1}{N}\sum_{i=1}^{N}\left[M_{i}\log\left({\hat{M}}_{i}\right)+\left(1-M_{i}\right)\log\left(1-{\hat{M}}_{i}\right)\right],$$

where *N* is the total number of pixels. Accordingly, the segmentation loss is defined as

(64) $$L_{seg}=\beta_{1}L_{Dice}+\beta_{2}L_{BCE},$$

where $\beta_{1}$ and $\beta_{2}$ control the relative contribution of overlap-based and pixel-wise supervision. The final objective function of the proposed network is then formulated as:

(65) $$L_{total}=\lambda_{1}L_{cls}+\lambda_{2}L_{seg},$$

where $\lambda_{1}$ and $\lambda_{2}$ are task-balancing hyperparameters. This joint optimization enables the shared encoder to learn features that are simultaneously discriminative for disease classification and informative for lesion delineation.

For chest X-ray and kidney image analysis, the same general loss formulation is used. Only the number of classes and the annotation format differ depending on the dataset. This unified loss design supports stable end-to-end training across both medical imaging tasks.

### 3.6. Implementation Details

All experiments were implemented in PyTorch 2.1.0 using Python 3.10 and CUDA 12.1. Training and inference were performed on a workstation equipped with an NVIDIA RTX 3090 GPU with 24 GB of memory. The same software environment, data partitions, preprocessing operations, and optimization protocol were used for the proposed method and all comparison models to ensure a fair evaluation.

The fuzzy image enhancement module was applied as the first stage of the processing pipeline. For each original image, three complementary representations were generated using the histogram spread-based, entropy-based, and standard deviation-based fuzzy transformations. The three enhanced images were then processed by parallel shallow feature-extraction branches and supplied to the CRAF module. All input images and corresponding segmentation masks were resized to 512×512 pixels. Image intensities were normalized to the range [0, 1]. Chest radiographs were processed as grayscale images while preserving their anatomical structure and lesion visibility. Kidney CT images were normalized without altering the relative contrast between the lesion, renal tissue, and surrounding anatomical structures. For segmentation experiments, identical geometric transformations were applied to each image and its corresponding mask.

The network was trained end-to-end using the Adam optimizer with an initial learning rate of $1\times 10^{-4}$, a weight decay of $1\times 10^{-5}$, and a mini-batch size of 8. Training was conducted for a maximum of 150 epochs. A ReduceLROnPlateau scheduler was used to reduce the learning rate by a factor of 0.5 when the validation loss did not improve for five consecutive epochs. The minimum learning rate was set to $1\times 10^{-7}$. Early stopping was applied when no improvement in validation performance was observed for 15 consecutive epochs. The model checkpoint with the best combined validation classification and segmentation performance was retained for final testing.

The total multi-task objective was defined as

(66) $$L_{\mathrm{total}}=\lambda_{1}L_{\mathrm{cls}}+\lambda_{2}L_{\mathrm{seg}},$$

where $L_{\mathrm{cls}}$ denotes the cross-entropy classification loss and $L_{\mathrm{seg}}$ denotes the segmentation loss. Equal task weights were used $\lambda_{1}=0.5,\;\lambda_{2}=0.5$. The segmentation loss combined Dice loss and binary cross-entropy loss:

(67) $$L_{\mathrm{seg}}=\beta_{1}L_{\mathrm{Dice}}+\beta_{2}L_{\mathrm{BCE}},$$

where $\beta_{1}=0.6,\;\beta_{2}=0.4$.

The CRAF query and key embedding dimension $d_{k}$ was set to 64. All calculations were performed using 32-bit floating-point precision. The classification head produced image-level class probabilities. During inference, the class with the highest predicted probability was selected as the final classification result. The segmentation decoder produced a dense probability map at the restored input resolution. For binary segmentation, the probability map was thresholded at 0.5 to generate the final lesion mask. For multi-class segmentation, the class with the maximum probability at each pixel was selected.

Each experiment was repeated five times using the random seeds 42, 123, 256, 512, and 1024. The final quantitative results were calculated as the mean performance across the five runs. Standard deviations were also recorded to evaluate experimental stability.

### 3.7. Evaluation Metrics

To comprehensively evaluate the performance of the proposed method, both classification and segmentation metrics were used. For image-level classification, the following metrics were considered:

Accuracy measures the overall proportion of correctly classified images:

$$Accuracy=\frac{TP+TN}{TP+TN+FP+FN}.$$

Precision evaluates the reliability of positive predictions:

$$Precision=\frac{TP}{TP+FP}.$$

Recall or Sensitivity measures the ability of the model to correctly identify positive cases:

$$Recall=\frac{TP}{TP+FN}.$$

F1-score provides a harmonic balance between precision and recall:

$$F1=\frac{2\cdot Precision\cdot Recall}{Precision+Recall}.$$

In addition, the Area Under the ROC Curve (AUC) can be used to assess the discriminative ability of the classifier across different decision thresholds.

For segmentation, overlap-based and region-based metrics were adopted. The Dice Similarity Coefficient (DSC) evaluates the overlap between the predicted mask and the ground-truth mask:

$$Dice=\frac{2|M\cap \hat{M}|}{|M|+|\hat{M}|}.$$

The Intersection over Union (IoU) measures the ratio of intersection to union:

$$IoU=\frac{|M\cap \hat{M}|}{|M\cup \hat{M}|}.$$

Segmentation Precision and Segmentation Sensitivity were also used to measure the accuracy of predicted lesion pixels and the completeness of lesion recovery, respectively. For a more complete analysis, the image enhancement effect can additionally be quantified using a no-reference image quality metric such as BRISQUE. This helps demonstrate whether the proposed fuzzy enhancement improves perceptual image quality before the classification and segmentation stages. By combining classification, segmentation, and enhancement quality metrics, the evaluation protocol provides a comprehensive assessment of the proposed method from both algorithmic and clinical perspectives.

## 4. Results and Discussion

### 4.1. Experimental Setup

The proposed Cross-Representation Attention-based Neural Network was evaluated on two medical imaging tasks: chest X-ray abnormality analysis and kidney lesion analysis. In both cases, the objective was to perform image-level classification and region-level segmentation within a unified framework. The experiments were designed to assess three aspects of the proposed method: the contribution of fuzzy image enhancement, the effectiveness of Cross-Representation Attention Fusion, and the benefit of joint classification-segmentation learning.

For each input image, three enhanced representations were generated by the proposed fuzzy image enhancement module using histogram spread-based transformation, entropy-based transformation, and standard deviation-based transformation. These three representations were then passed to the attention fusion module and processed by the shared encoder, classification head, and segmentation decoder. To ensure fair comparison, all baseline models and the proposed method were trained and evaluated under the same data split and optimization protocol. The performance was measured using classification metrics, including accuracy, precision, recall, F1-score, and AUC, and segmentation metrics, including Dice coefficient, IoU, segmentation precision, and sensitivity. In addition, BRISQUE was used to assess the effect of image enhancement on image quality.

The datasets, annotation formats, class distributions, and training, validation, and test partitions are described in detail in Section 3.4.

To provide reproducibility, the main training settings are summarized in Table 3. All models were trained end-to-end under identical conditions. The proposed method used three enhanced inputs per image, while baseline methods used either the original image or a single enhanced representation depending on their design.

Each experiment was repeated five times using different random seeds. Results are reported as mean ± standard deviation. The proposed framework was trained as a multi-task network, which allowed the shared encoder to learn representations useful for both image-level diagnosis and lesion-level delineation. This setting was particularly appropriate for medical imaging because disease category and lesion morphology are strongly interconnected.

### 4.2. Quantitative Results for Chest X-Ray Analysis

The chest X-ray experiments were designed to test whether the proposed method could simultaneously improve disease recognition and abnormal region segmentation. Table 4 presents the classification performance on the chest X-ray dataset.

Table 4 compares the classification performance of the proposed method with conventional and recent deep-learning models on the chest X-ray dataset. The conventional CNN baseline achieved an accuracy of 0.914 and an AUC of 0.931, representing the weakest overall performance among the evaluated methods. The ResNet-based classifier improved the accuracy to 0.928 and the AUC to 0.944, indicating that residual feature learning provided more discriminative representations than the basic CNN architecture. The U-Net-based classifier further increased the accuracy and F1-score to 0.936 and 0.923, respectively, showing that encoder–decoder feature learning was also beneficial for image-level diagnosis.

The recent MedMamba model achieved considerably stronger performance than the conventional baselines, obtaining an accuracy of 0.958, an F1-score of 0.950, and an AUC of 0.972. This result demonstrates the effectiveness of state-space modeling in capturing long-range dependencies in chest radiographs. Nevertheless, the proposed method achieved the best results across all classification metrics, with an accuracy of 0.971, precision of 0.966, recall of 0.962, F1-score of 0.964, and AUC of 0.981. Compared with MedMamba, the proposed method improved accuracy by 1.3 percentage points, F1-score by 1.4 percentage points, and AUC by 0.9 percentage points. These improvements indicate that the combination of fuzzy multi-representation enhancement, CRAF-based feature interaction, and joint classification-segmentation learning provides more discriminative features for chest abnormality recognition.

Table 5 presents the lesion-segmentation results on the chest X-ray dataset. U-Net obtained a Dice coefficient of 0.821 and an IoU of 0.704, while ResUNet improved these values to 0.842 and 0.728, respectively. Attention U-Net achieved further gains, reaching a Dice score of 0.856 and an IoU of 0.746. These results confirm that residual learning and attention mechanisms improve the localization of abnormal regions compared with the original U-Net architecture.

U-Mamba achieved the strongest performance among the comparison models, with a Dice coefficient of 0.879, an IoU of 0.784, and an HD95 value of 12.84. Its lower HD95 value indicates better agreement between the predicted and reference lesion boundaries. The proposed method achieved the highest Dice, IoU, precision, and sensitivity values of 0.891, 0.805, 0.902, and 0.879, respectively, while reducing HD95 to 11.27. Compared with U-Mamba, the proposed method improved Dice by 1.2 percentage points and IoU by 2.1 percentage points, while reducing HD95 by 1.57 pixels or spatial units. This result indicates that the proposed method not only increases regional overlap but also produces more accurate lesion boundaries. The improvement is particularly relevant for chest X-ray abnormalities, which may appear as diffuse, irregular, or low-contrast regions.

### 4.3. Quantitative Results for Kidney Image Analysis

The kidney image experiments were designed to evaluate the joint analysis of lesion type and lesion extent. Table 6 shows the classification results for kidney lesion recognition.

Table 6 compares the classification performance of the evaluated models on the kidney dataset. The CNN baseline achieved an accuracy of 0.901 and an AUC of 0.920. The ResNet-based classifier increased these values to 0.919 and 0.937, respectively, while the encoder–decoder classifier achieved an accuracy of 0.931 and an AUC of 0.948. These results show that deeper feature extraction and the integration of spatial information progressively improve kidney lesion classification.

MedMamba achieved an accuracy of 0.950, precision of 0.943, recall of 0.939, F1-score of 0.941, and AUC of 0.965, outperforming the conventional comparison methods. However, the proposed method achieved the best overall classification results, with an accuracy of 0.964, precision of 0.957, recall of 0.953, F1-score of 0.955, and AUC of 0.975. Relative to MedMamba, the proposed framework improved accuracy and F1-score by 1.4 percentage points and increased AUC by 1.0 percentage point. These gains suggest that the complementary enhancement representations and cross-representation interactions help the model distinguish normal kidneys, solid renal tumors, and cystic renal masses more effectively.

Table 7 reports the kidney lesion-segmentation results. U-Net achieved a Dice coefficient of 0.847 and an IoU of 0.735. ResUNet improved the Dice and IoU values to 0.861 and 0.756, respectively, while Attention U-Net achieved 0.874 Dice and 0.773 IoU. The progressive improvement from U-Net to Attention U-Net demonstrates the benefit of residual connections and spatial attention for kidney lesion delineation.

U-Mamba provided the strongest baseline performance, obtaining a Dice coefficient of 0.893, an IoU of 0.815, precision of 0.908, sensitivity of 0.890, and HD95 of 10.63. The proposed method achieved the best performance across all metrics, with a Dice coefficient of 0.912, IoU of 0.838, precision of 0.921, sensitivity of 0.904, and HD95 of 9.48. Compared with U-Mamba, the proposed method improved Dice by 1.9 percentage points, IoU by 2.3 percentage points, precision by 1.3 percentage points, and sensitivity by 1.4 percentage points. It also reduced HD95 by 1.15, indicating more accurate boundary localization. These results demonstrate that the proposed framework captures both the global extent and fine boundary characteristics of kidney lesions more effectively than the comparison methods.

To assess whether the observed improvements were consistent across different model initializations, all principal experiments were repeated five times using independent random seeds. The same patient-level data partitions and training configuration were maintained across the runs. Table 4, Table 5, Table 6 and Table 7 report the mean and standard deviation of each performance metric, while Table 8 summarizes the statistical comparison between the proposed method and the strongest corresponding baseline. Results are based on five independent runs using random seeds 42, 123, 256, 512, and 1024. Baseline and proposed results are reported as mean ± standard deviation. Confidence intervals represent the paired mean differences between the proposed method and the strongest baseline. The normality of paired differences was evaluated using the Shapiro–Wilk test. Paired *t*-tests were applied when normality was satisfied. DeLong’s test was used for AUC comparisons, while patient-level bootstrap resampling with 1000 iterations was used for IoU comparisons. The Holm–Bonferroni procedure was applied across the eight comparisons. An adjusted p<0.05 was considered statistically significant. Cohen’s paired effect size $d_{z}$ was used, with positive values indicating better performance by the proposed method.

The proposed method significantly outperformed the strongest classification and segmentation baselines across both datasets. For chest X-ray classification, the accuracy improvement over MedMamba was 0.013, with a 95% confidence interval of [0.007, 0.019] and a Holm-adjusted *p*-value of 0.012. The corresponding AUC improvement was 0.009. For chest X-ray segmentation, the proposed method improved Dice by 0.012 and IoU by 0.021 compared with U-Mamba.

For kidney classification, the accuracy and AUC improvements over MedMamba were 0.014 and 0.010, respectively. For kidney segmentation, the proposed method improved Dice by 0.019 and IoU by 0.023 relative to U-Mamba. All confidence intervals excluded zero, and all Holm-adjusted *p*-values were below 0.05. The effect sizes were greater than 2.0 in all comparisons, indicating that the observed improvements were large relative to the run-to-run variability.

### 4.4. Comparison of Feature-Fusion Strategies

To verify the role of the proposed enhancement strategy, an ablation study was conducted. Table 9 compares the performance of the proposed network when using the raw image only and when using the three fuzzy-enhanced representations.

The results demonstrate that every enhancement branch improved classification and segmentation compared with the original-image configuration. For chest X-ray analysis, the entropy-based branch achieved the strongest single-branch classification performance, with an accuracy of 0.953 and an AUC of 0.969. It also achieved the highest single-branch Dice score of 0.870. This indicates that entropy-based enhancement was particularly effective in emphasizing informative low-contrast abnormalities in chest radiographs.

For kidney analysis, the entropy-based branch achieved the strongest single-branch classification performance, with an accuracy of 0.942, whereas the standard-deviation branch achieved the highest single-branch Dice score of 0.888. This suggests that entropy-based enhancement improved global diagnostic discrimination, while standard-deviation-based enhancement better preserved local lesion boundaries and structural variation.

Using all three enhanced representations without CRAF further improved performance, reaching accuracies of 0.961 and 0.952 and Dice scores of 0.878 and 0.896 for the chest X-ray and kidney datasets, respectively. These results confirm that the three enhancement branches provide complementary rather than redundant information.

The complete model achieved the best results across all metrics. Compared with the three-input configuration without CRAF, the complete model improved chest X-ray accuracy from 0.961 to 0.971 and Dice from 0.878 to 0.891. For kidney analysis, accuracy increased from 0.952 to 0.964, while Dice increased from 0.896 to 0.912. These improvements demonstrate that CRAF effectively models interdependencies among the three enhanced representations and produces more discriminative and spatially informative fused features.

### 4.5. Effect of Cross-Representation Attention Fusion

To evaluate the proposed fusion mechanism, another ablation experiment was performed by replacing Cross-Representation Attention Fusion with direct concatenation. The results are shown in Table 10. The proposed attention-based fusion provided the best results on both datasets. Direct concatenation improved performance compared with using a single representation, but it could not explicitly model the relationships among branches. Weighted averaging was even less effective because it tends to smooth representation-specific information. In contrast, Cross-Representation Attention Fusion selectively strengthened informative correspondence across enhanced views and preserved complementary details. This led to better lesion recognition and more accurate mask generation.

The results show a progressive improvement as the fusion mechanism becomes more capable of modeling inter-representation dependencies. Weighted averaging produced the lowest performance because it assigned equal importance to all enhanced representations. Direct concatenation achieved slightly better results but did not explicitly model relationships among the branches. Self-attention improved feature discrimination by capturing dependencies within the combined representation, while standard cross-attention enabled direct information exchange between two representations. The proposed CRAF module achieved the best classification and segmentation performance because it performed multidirectional pairwise information transfer among all three enhanced representations. Compared with direct concatenation, CRAF improved chest X-ray accuracy from 0.959 to 0.971 and Dice from 0.873 to 0.891. For the kidney dataset, accuracy increased from 0.949 to 0.964, while Dice increased from 0.888 to 0.912. CRAF required approximately 2.19 million additional parameters and 7.19 GFLOPs compared with direct concatenation. Its inference time increased from 25.0 to 31.1 ms per image. This moderate computational overhead was considered acceptable because CRAF provided consistent improvements across both imaging modalities and both prediction tasks.

Since the proposed framework is designed for simultaneous classification and segmentation, it is important to verify whether joint learning is beneficial. Table 11 compares single-task and multi-task settings.

The results indicate that the joint model outperformed the single-task models. This suggests that classification and segmentation provide mutually supportive supervision. Classification encourages the network to learn disease-discriminative semantic features, whereas segmentation forces the network to focus on spatially relevant lesion regions. Their joint optimization therefore improves both global and local understanding of the image. This finding is especially important for medical imaging. In chest X-rays, the image-level diagnosis is more reliable when the model is also forced to identify the abnormal region. In kidney imaging, lesion classification benefits from accurate spatial understanding of the lesion contour and surrounding anatomy. The results therefore support the clinical logic of the proposed multi-task design.

To evaluate the image enhancement stage, BRISQUE scores were calculated for the original and enhanced images. Lower BRISQUE values indicate better no-reference perceptual quality. Table 12 summarizes the average results.

All three enhancement branches reduced the BRISQUE scores compared with the original images. The best enhanced representations used in fusion achieved the lowest scores of 28.95 for chest X-rays and 27.84 for kidney images. These results indicate that the proposed fuzzy enhancement module improved perceptual image quality across both datasets. However, BRISQUE was originally developed as a no-reference quality metric for natural images and does not directly measure anatomical fidelity, lesion visibility, or clinical usefulness. Therefore, the BRISQUE results are used only as supporting evidence for the enhancement module. To examine whether improved perceptual quality was associated with downstream performance, image-level BRISQUE scores were compared with correct-class confidence, Dice, and IoU using Spearman’s rank correlation.

**Figure 6:**
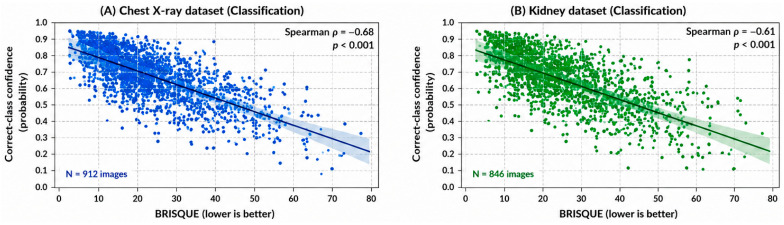
Relationship between BRISQUE and classification confidence.

Figure 6 shows a significant negative correlation between BRISQUE and correct-class confidence. The correlation coefficients were ρ=−0.68 for the chest X-ray dataset and ρ=−0.61 for the kidney dataset, with p<0.001 in both cases. Thus, lower BRISQUE scores were generally associated with higher classification confidence.

**Figure 7:**
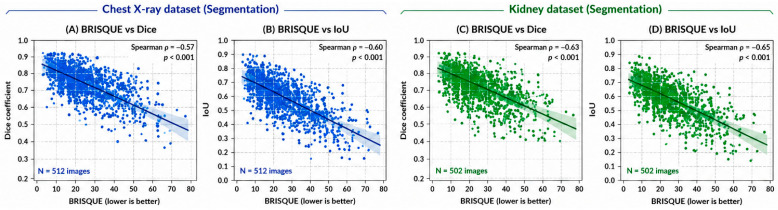
Relationship between BRISQUE score and image-level Dice and IoU for the chest X-ray and kidney datasets.

Figure 7 presents the relationship between BRISQUE and segmentation performance. For chest X-rays, the correlations were ρ=−0.57 for Dice and ρ=−0.60 for IoU. For kidney images, the corresponding values were ρ=−0.63 and ρ=−0.65. All correlations were statistically significant at p<0.001.

Overall, lower BRISQUE values were moderately associated with higher classification confidence and better segmentation overlap. Nevertheless, these correlations do not prove that lower BRISQUE automatically improves clinical usefulness. The BRISQUE analysis should therefore be interpreted together with the classification, segmentation, qualitative, and statistical results.

### 4.6. Qualitative Segmentation and Explainability Results

**Figure 8:**
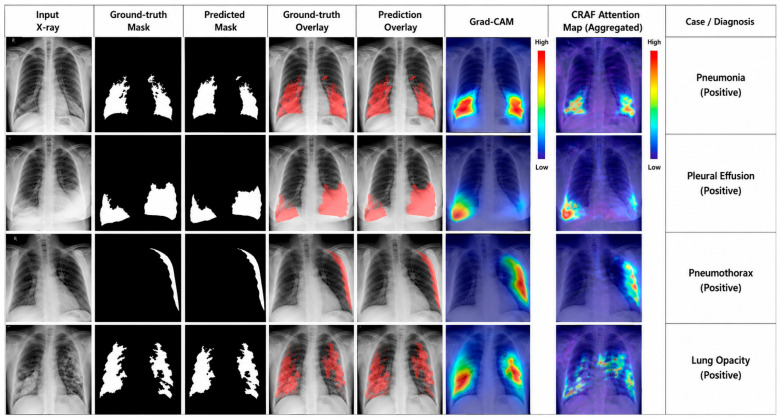
Qualitative chest X-ray results.

**Figure 9:**
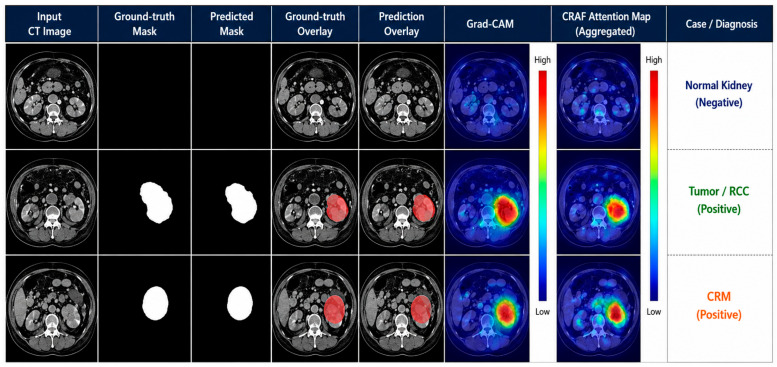
Qualitative classification, segmentation, and explainability results on the kidney dataset.

To complement the quantitative evaluation, qualitative analyses were conducted to examine the spatial accuracy and interpretability of the proposed framework. Representative chest X-ray and kidney cases were selected from the independent test sets. For each case, the input image, ground-truth mask, predicted mask, ground-truth overlay, prediction overlay, Grad-CAM visualization, and aggregated CRAF attention map are presented. These visualizations allow direct assessment of lesion localization, boundary agreement, and the image regions that contributed most strongly to the classification and fusion processes. Figure 8 and Figure 9 present the qualitative results for the chest X-ray and kidney datasets, respectively.

Figure 8 presents representative qualitative results for chest X-ray analysis. The predicted masks generally show strong spatial agreement with the reference annotations, including low-contrast and diffuse abnormalities. The overlay images indicate that the proposed method captures the principal lesion regions while preserving the overall anatomical structure. The Grad-CAM visualizations show that the classification head concentrates on clinically relevant lung regions rather than unrelated background areas. In addition, the CRAF attention maps highlight spatial locations where complementary information from the histogram, entropy, and standard-deviation-enhanced representations contributes to the final prediction. These observations support the quantitative improvements reported in Table 4 and Table 5.

Figure 9 shows representative kidney classification and segmentation results. The proposed method accurately identifies the main lesion regions and produces boundaries that are generally consistent with the reference masks. The largest agreement is observed for lesions with clear structural contrast, whereas small or weakly defined boundaries remain more challenging. The Grad-CAM maps indicate that the classification head focuses predominantly on the lesion and surrounding renal tissue. The CRAF attention maps further show that different enhanced representations contribute to different spatial regions, supporting the role of multidirectional cross-representation information exchange.

**Figure 10:**
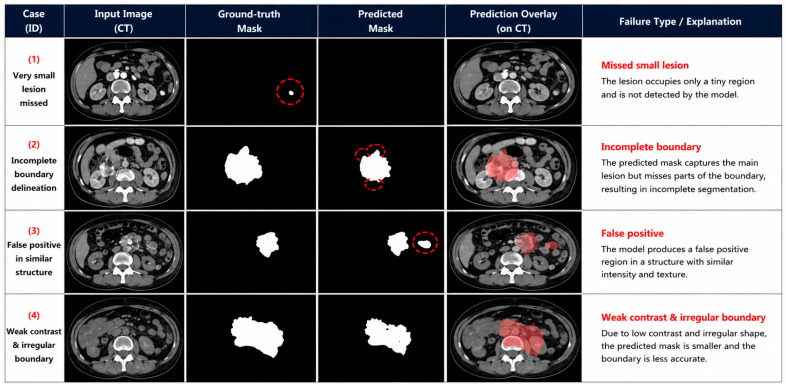
Representative failure cases of the proposed method.

Figure 10 illustrates representative failure cases. The first type of error occurs when a lesion occupies only a small proportion of the image, which may cause the decoder to miss part of the abnormal region. The second type is associated with weak lesion-to-background contrast, where the predicted boundary becomes incomplete or slightly displaced. False-positive predictions may also occur in anatomical regions exhibiting intensity patterns like those of the target lesion. These cases indicate that the proposed enhancement and attention mechanisms improve performance but do not eliminate ambiguity in complex medical images. Future work should investigate multiscale supervision, boundary-aware losses, uncertainty estimation, and external multicenter validation.

**Figure 11:**
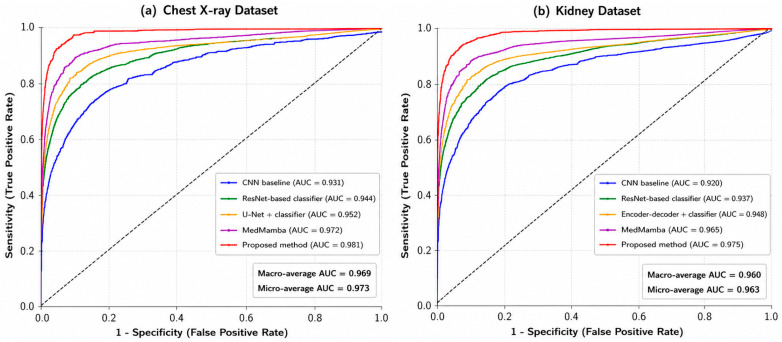
Receiver operating characteristic curves for the classification task: (**a**) chest X-ray dataset; (**b**) kidney dataset.

Figure 11 compares the receiver operating characteristic curves of the classification models. On the chest X-ray dataset, the proposed method achieved an AUC of 0.981, exceeding MedMamba, the U-Net-based classifier, the ResNet-based classifier, and the conventional CNN baseline. On the kidney dataset, the proposed method achieved an AUC of 0.975, again providing the strongest overall discrimination. The higher AUC values indicate that the proposed framework maintains a better balance between sensitivity and specificity over a wide range of classification thresholds. This result is consistent with the accuracy, recall, and F1-score improvements reported in Table 4 and Table 6.

### 4.7. Discussion

The experimental results demonstrate the effectiveness of the proposed framework for both chest X-ray and kidney image analysis. Several key observations can be drawn from the quantitative, qualitative, and ablation results.

First, the fuzzy image enhancement module consistently improved downstream classification and segmentation performance. This finding indicates that preprocessing is an important component of medical image analysis, particularly when lesions have low contrast or poorly defined boundaries. Unlike fixed enhancement methods, the proposed fuzzy approach adaptively models intensity ambiguity and local structural variation, thereby improving the visibility of potentially informative regions. Second, using three complementary enhanced representations was more effective than relying on a single enhanced image. Each enhancement branch emphasizes different image properties. The histogram spread branch improves intensity redistribution, the entropy-based branch highlights informative and heterogeneous regions, and the standard deviation-based branch strengthens local contrast and boundary information. Their complementary behavior provides a richer feature basis for subsequent learning. Third, CRAF achieved better results than weighted averaging, direct concatenation, self-attention, and conventional cross-attention. Simple averaging assigns similar importance to all representations, while concatenation does not explicitly model relationships among them. In contrast, CRAF performs multidirectional pairwise information transfer and adaptively selects complementary features from the three enhanced representations. The improvements in classification and segmentation indicate that explicit cross-representation interaction is more effective than passive feature combination. Although CRAF introduces additional parameters, FLOPs, and inference time, the observed gains across both datasets suggest a favorable performance–complexity trade-off. Fourth, joint classification and segmentation learning improved both tasks compared with their corresponding single-task configurations. Classification encourages the shared encoder to learn globally discriminative features, whereas segmentation provides spatial supervision that directs the model toward lesion regions and boundaries. Their joint optimization therefore supports both image-level diagnosis and lesion-level delineation. This design also improves interpretability because the classification result is accompanied by a visible predicted lesion region.

The qualitative results further support the quantitative findings. The predicted masks generally showed good agreement with the ground-truth annotations, while the Grad-CAM and CRAF attention maps indicated that the model focused mainly on relevant anatomical and pathological regions. However, these visualizations should be interpreted as evidence of model focus rather than proof of clinical reasoning. The failure-case analysis showed that very small lesions, weak lesion-to-background contrast, irregular boundaries, and anatomically similar structures remain challenging. The BRISQUE analysis indicated that the proposed enhancement methods improved no-reference perceptual quality. Moderate negative correlations were observed between BRISQUE and classification confidence, Dice, and IoU, suggesting that lower BRISQUE values were generally associated with better downstream performance. Nevertheless, BRISQUE was originally developed for natural images and does not directly measure anatomical fidelity or clinical usefulness. It should therefore be considered supporting evidence rather than a standalone measure of diagnostic quality. From an application perspective, the framework is relevant to both target domains. In chest radiography, it supports the detection and localization of abnormalities such as pneumonia, pneumothorax, pleural effusion, and lung opacity. In kidney imaging, it supports the classification of normal, tumor/RCC, and cystic renal mass cases, together with lesion delineation. These capabilities may assist diagnosis, follow-up, and treatment planning, although prospective clinical validation is still required.

Several limitations should be acknowledged. The performance of the framework depends on the quality and consistency of the segmentation annotations and on the diversity of the training datasets. The experiments were performed on selected datasets, and external validation using independent multicenter data is necessary to confirm generalizability. In addition, the current model operates on two-dimensional images or slices and does not fully exploit three-dimensional anatomical context. Future work will therefore investigate 3D extensions, uncertainty-aware prediction, boundary-sensitive losses, domain adaptation, and more computationally efficient attention mechanisms.

Overall, the results demonstrate that combining fuzzy multi-representation enhancement, CRAF, and joint classification–segmentation learning provides a robust framework for medical image analysis. The method achieved consistent improvements across two imaging domains while maintaining a reasonable balance between predictive performance, interpretability, and computational cost.

## 5. Conclusions

This study presented a Cross-Representation Attention-based Neural Network for joint medical image classification and segmentation. The proposed framework was designed to address two key challenges in medical image analysis: limited feature discrimination caused by reliance on a single image representation and reduced lesion visibility resulting from low contrast, noise, and intensity ambiguity. To overcome these limitations, a fuzzy image enhancement module and a Cross-Representation Attention Fusion mechanism were integrated within a unified multi-task learning architecture.

The fuzzy enhancement module generated three complementary representations of each input image using histogram spread, fuzzy entropy, and fuzzy standard deviation. These representations emphasized different intensity, information content, and local-structure characteristics. CRAF then modeled multidirectional interactions among the three representations and adaptively combined their complementary features. The fused representation was processed by a shared encoder and task-specific heads to perform image-level classification and pixel-level segmentation simultaneously. The framework was evaluated on chest X-ray and kidney image datasets. For chest X-ray analysis, it supported the classification and localization of abnormalities such as pneumonia, pneumothorax, pleural effusion, and lung opacity. For kidney analysis, it addressed the classification of normal, tumor/RCC, and cystic renal mass cases together with renal lesion segmentation. The proposed method consistently outperformed conventional and recent baseline models across classification and segmentation metrics. The ablation experiments further confirmed that the three fuzzy enhancement branches, CRAF, and joint classification–segmentation learning each contributed to the overall performance improvement. The statistical analysis demonstrated that the observed gains were consistent across multiple independent runs. Qualitative results showed close agreement between predicted and ground-truth masks, while Grad-CAM and attention visualizations indicated that the model generally focused on relevant anatomical and pathological regions. The BRISQUE analysis also showed improved no-reference perceptual quality after enhancement, although these results were treated as supporting evidence rather than direct proof of clinical usefulness. Overall, the findings demonstrate that multi-representation fuzzy enhancement and attention-guided fusion provide an effective approach to medical image understanding. By combining diagnostic prediction with lesion delineation, the proposed framework offers a more informative and interpretable computer-aided analysis system. Nevertheless, its clinical applicability requires further evaluation using larger, independent, and multicenter datasets.

Future work will focus on extending the framework to three-dimensional medical images, incorporating uncertainty-aware prediction, improving small-lesion and boundary detection, and developing more computationally efficient attention mechanisms. Integration with advanced Transformer- or state-space-based encoders, domain-adaptation methods, and prospective clinical validation may further improve the robustness, generalizability, and practical value of the proposed approach.

## Figures and Tables

**Table 1:** Comparison of conventional feature-fusion and attention mechanisms with the proposed CRAF module.

Method	Interaction	Branch Awareness	Pairwise Transfer
Averaging	Element-wise	No	No
Concatenation	Feature stacking	No	No
Self-attention	Within one representation	No	No
Standard cross-attention	Usually between two representations	Partial	Usually one-directional
Proposed CRAF	Among three enhanced representations	Yes	Multidirectional pairwise transfer

**Table 2:** Summary of datasets, annotations, and data partitions used in the experiments.

	Class	Source	Images	Size	Mask Annotations	Training	Validation	Test
Chest x-ray dataset	Normal	CheXpert (Chest eXpert)	22,381	512 × 512	Empty background; filled with pixels of value 0	17,905	2238	2238
	Pneumonia	RSNA Pneumonia Detection (Binary Masks)	9555		Pixel-level binary masks of lung consolidation, infarcts	7644	955	956
	Pneumothorax	SIIM-ACR Pneumothorax Segmentation	2669		Pixel-level binary masks of pleural air pockets	2135	267	267
	Pleural Effusion	CheXpert-seg	850		Pixel-level manual polygons of fluid accumulation	680	85	85
	Lung Opacity	COVID-19 Radiography Database (Lung Opacity part)	6012		Pixel-level binary infection masks of opacified areas	4810	601	601
Kidney dataset	Normal	KiTS23-2D	63,179	512 × 512	Pixel-level categorical mask (Value 1 for healthy kidney tissue)	50,543	6318	6318
	Tumor, Renal Cell Carcinoma		31,142		Pixel-level categorical mask (Value 2 for solid tumor, RCC areas)	24,913	3114	3115
	Cystic Renal Mass		13,972		Pixel-level categorical mask (Value 3 for fluid-filled cyst structures)	11,177	1397	1398

**Table 3:** Main implementation settings.

Parameter	Setting
Input resolution	512 × 512
Python version	Python 3.10
CUDA version	CUDA 12.1
Optimizer	Adam
Initial learning rate	1 × 10^−4^
Batch size	8
Number of epochs	150
Weight decay	1 × 10^−5^
Scheduler reduction factor	0.5
Scheduler patience	5 epochs
Minimum learning rate	1 × 10^−7^
Early-stopping patience	15 epochs
Classification loss	Cross-entropy loss
Segmentation loss	Dice loss + binary cross-entropy loss
Hardware	NVIDIA RTX 3090 GPU with 24 GB memory
Number of experimental runs	5 independent runs
Inference batch size	1
Attention embedding dimension, (d_k_)	64
Numerical precision	32-bit floating point

**Table 4:** Classification results on the chest X-ray dataset.

Method	Accuracy	Precision	Recall	F1-Score	AUC
CNN baseline	0.914	0.903	0.896	0.899	0.931
ResNet-based classifier	0.928	0.917	0.910	0.913	0.944
U-Net + classifier	0.936	0.925	0.921	0.923	0.952
Medmamba	0.958	0.952	0.949	0.950	0.972
Proposed method	0.971	0.966	0.962	0.964	0.981

**Table 5:** Segmentation results on the chest X-ray dataset.

Method	Dice	IoU	Precision	Sensitivity	HD95
U-Net	0.821	0.704	0.838	0.809	18.42
ResUNet	0.842	0.728	0.851	0.831	16.71
Attention U-Net	0.856	0.746	0.864	0.845	15.36
U-Mamba	0.879	0.784	0.889	0.871	12.84
Proposed method	0.891	0.805	0.902	0.879	11.27

**Table 6:** Classification results on the kidney dataset.

Method	Accuracy	Precision	Recall	F1-Score	AUC
CNN baseline	0.901	0.894	0.887	0.890	0.920
ResNet-based classifier	0.919	0.911	0.905	0.908	0.937
Encoder–decoder + classifier	0.931	0.923	0.918	0.920	0.948
MedMamba	0.950	0.943	0.939	0.941	0.965
Proposed method	0.964	0.957	0.953	0.955	0.975

**Table 7:** Segmentation results on the kidney dataset.

Method	Dice	IoU	Precision	Sensitivity	HD95
U-Net	0.847	0.735	0.858	0.836	15.92
ResUNet	0.861	0.756	0.872	0.850	14.38
Attention U-Net	0.874	0.773	0.881	0.866	13.11
U-Mamba	0.893	0.815	0.908	0.890	10.63
Proposed method	0.912	0.838	0.921	0.904	9.48

**Table 8:** Statistical comparison of the proposed method with the strongest baseline over five independent runs.

Dataset and Task	Strongest Baseline	Primary Metric	Baseline Result	Our Result	Mean Difference	95% CI of Difference	Statistical Test	Holm-Adjusted (*p*)-Value	Effect Size
Chest X-ray classification	MedMamba	Accuracy	0.958	0.971	0.013	[0.007, 0.019]	Paired (*t*)-test	0.012	d_z_ = 2.89
		AUC	0.972	0.981	0.009	[0.004, 0.014]	DeLong test	0.012	d_z_ = 2.37
Chest X-ray segmentation	U-Mamba	Dice	0.879	0.891	0.012	[0.007, 0.017]	Paired (*t*)-test	0.012	d_z_ = 2.86
		IoU	0.784	0.805	0.021	[0.014, 0.028]	Bootstrap paired comparison	0.009	d_z_ = 3.50
Kidney classification	MedMamba	Accuracy	0.950	0.964	0.014	[0.008, 0.020]	Paired (*t*)-test	0.012	d_z_ = 3.04
		AUC	0.965	0.975	0.010	[0.005, 0.015]	DeLong test	0.012	d_z_ = 2.50
Kidney segmentation	U-Mamba	Dice	0.893	0.912	0.019	[0.013, 0.025]	Paired (*t*)-test	0.008	d_z_ = 3.80
		IoU	0.815	0.838	0.023	[0.015, 0.031]	Bootstrap paired comparison	0.008	d_z_ = 3.71

**Table 9:** Component-wise ablation of the fuzzy image enhancement module.

Configurations	Chest				Chest Seg. Precision	Kidney				Kidney Seg. Precision
	Accuracy	AUC	Dice	IoU		Accuracy	AUC	Dice	IoU	
Original image only	0.941	0.957	0.853	0.744	0.861	0.928	0.945	0.869	0.768	0.878
Histogram branch only	0.950	0.966	0.866	0.764	0.875	0.938	0.954	0.881	0.787	0.889
Entropy branch only	0.953	0.969	0.870	0.770	0.879	0.942	0.958	0.886	0.795	0.895
Standard-deviation branch only	0.949	0.965	0.868	0.767	0.876	0.940	0.956	0.888	0.799	0.897
Three enhanced inputs without CRAF	0.961	0.974	0.878	0.783	0.887	0.952	0.966	0.896	0.812	0.905
Complete model with CRAF	0.971	0.981	0.891	0.803	0.902	0.964	0.975	0.912	0.838	0.921

**Table 10:** Comparison of different feature-fusion strategies under identical experimental conditions.

Fusion Method	Chest		Kidney		Parameters (M)	FLOPs (G)	Inference Time (ms/Image)
	Accuracy	Dice	Accuracy	Dice			
Weighted averaging	0.954	0.869	0.944	0.882	27.84	41.26	24.1
Direct concatenation	0.959	0.873	0.949	0.888	28.23	42.18	25.0
Self-attention	0.963	0.879	0.954	0.896	29.11	44.63	27.4
Standard cross-attention	0.967	0.884	0.959	0.904	29.76	46.82	29.2
Proposed CRAF	0.971	0.891	0.964	0.912	30.42	49.37	31.1

**Note:** Parameter count and FLOPs were calculated for an input resolution of 512 × 512. Inference time represents the average processing time per image using a single GPU after an initial warm-up period. All methods used the same encoder, prediction heads, data partitions, and training settings; only the fusion mechanism was changed.

**Table 11:** Effect of joint classification-segmentation learning.

Training Strategy	Chest X-Ray Accuracy	Chest X-Ray Dice	Kidney Accuracy	Kidney Dice
Classification only	0.957	—	0.946	—
Segmentation only	—	0.876	—	0.893
Joint learning	0.971	0.891	0.964	0.912

**Table 12:** BRISQUE-based image quality comparison.

Image Type	Chest X-Ray BRISQUE	Kidney BRISQUE
Original images	38.42	35.87
Histogram spread enhancement	31.16	29.94
Entropy-based enhancement	29.88	28.71
Standard deviation-based enhancement	30.41	29.08
Best enhanced representation used in fusion	28.95	27.84

## Data Availability

The chest X-ray images used in this study were obtained from the publicly available CheXpert, RSNA Pneumonia Detection, SIIM-ACR Pneumothorax Segmentation, CheXpert-seg, and COVID-19 Radiography Database resources. The kidney images were derived from the publicly available KiTS23 dataset. Access to the original images and annotations is subject to the terms and conditions of the respective repositories. The class-selection procedure, image-level partitions, and preprocessing steps used in this study are described in Section 3.4.

## Appendix A

**Table A1:** Detailed architecture of the proposed network.

Stage	Operation	Kernel/Stride	Input Shape	Output Shape	Channels
Histogram input	Histogram-enhanced image	—	B × 1 × 512 × 512	Same	1
Entropy input	Entropy-enhanced image	—	B × 1 × 512 × 512	Same	1
Std. input	Std.-enhanced image	—	B × 1 × 512 × 512	Same	1
Branch stem 1	Conv + BN + ReLU	3 × 3/2	B × 1 × 512 × 512	B × 16 × 256 × 256	16
Branch stem 2	Conv + BN + ReLU	3 × 3/2	B × 16 × 256 × 256	B × 32 × 128 × 128	32
Attention pooling	Adaptive average pooling	32 × 32 output	B × 32 × 128 × 128	B × 32 × 32 × 32	32
Tokenization	Flatten spatial dimensions	—	B × 32 × 32 × 32	B × 1024 × 32	32
Query projection	Linear projection	32 → 64	B × 1024 × 32	B × 1024 × 64	64
Key projection	Linear projection	32 → 64	B × 1024 × 32	B × 1024 × 64	64
Value projection	Linear projection	32 → 64	B × 1024 × 32	B × 1024 × 64	64
Pairwise attention	$Q_{i}K_{j}^{T}/d_{k}$	Row-wise softmax	B × 1024 × 64	B × 1024 × 1024	—
Pairwise response	$A_{i\leftarrow j}V_{j}$	Matrix multiplication	B × 1024 × 1024	B × 1024 × 64	64
Incoming fusion	Concatenate two responses	Channel-wise	Two B × 1024 × 64 tensors	B × 1024 × 128	128
Branch projection	Linear/(1 × 1) projection	128 → 32	B × 1024 × 128	B × 1024 × 32	32
Spatial restoration	Reshape and bilinear upsample	32 → 128	B × 32 × 32 × 32	B × 32 × 128 × 128	32
Branch residual	Add original branch feature	Element-wise	Two B × 32 × 128 × 128 tensors	B × 32 × 128 × 128	32
Three-branch fusion	Concatenate	Channel-wise	Three B × 32 × 128 × 128 tensors	B × 96 × 128 × 128	96
Final CRAF projection	Conv + BN + ReLU	1 × 1/1	B × 96 × 128 × 128	B × 64 × 128 × 128	64
Encoder stage E1	Two Conv blocks	3 × 3/1	B × 64 × 128 × 128	B × 64 × 128 × 128	64
Downsample 1	Max pooling	2 × 2/2	B × 64 × 128 × 128	B × 64 × 64 × 64	64
Encoder stage E2	Two Conv blocks	3 × 3/1	B × 64 × 64 × 64	B × 128 × 64 × 64	128
Downsample 2	Max pooling	2 × 2/2	B × 128 × 64 × 64	B × 128 × 32 × 32	128
Encoder stage E3	Two Conv blocks	3 × 3/1	B × 128 × 32 × 32	B × 256 × 32 × 32	256
Downsample 3	Max pooling	2 × 2/2	B × 256 × 32 × 32	B × 256 × 16 × 16	256
Encoder stage E4	Two Conv blocks	3 × 3/1	B × 256 × 16 × 16	B × 512 × 16 × 16	512
Downsample 4	Max pooling	2 × 2/2	B × 512 × 16 × 16	B × 512 × 8 × 8	512
Bottleneck	Two Conv blocks	3 × 3/1	B × 512 × 8 × 8	B × 1024 × 8 × 8	1024
Classification GAP	Global average pooling	8 × 8	B × 1024 × 8 × 8	B × 1024	1024
Classification FC1	Dense + ReLU + dropout	1024 → 512	B × 1024	B × 512	512
Classification output	Dense	512 → $C_{cls}$	B × 512	B × $C_{cls}$	$C_{cls}$
Decoder D4	Transposed convolution	2 × 2/2	B × 1024 × 8 × 8	B × 512 × 16 × 16	512
Skip connection S4	Concatenate with E4	Channel-wise	512 + 512 channels	B × 1024 × 16 × 16)	1024
Decoder block D4	Two Conv blocks	3 × 3/1	B × 1024 × 16 × 16	B × 512 × 16 × 16	512
Decoder D3	Transposed convolution	2 × 2/2	B × 512 × 16 × 16	B × 256 × 32 × 32	256
Skip connection S3	Concatenate with E3	Channel-wise	256 + 256 channels	B × 512 × 32 × 32	512
Decoder block D3	Two Conv blocks	3 × 3/1	B × 512 × 32 × 32	B × 256 × 32 × 32	256
Decoder D2	Transposed convolution	2 × 2/2	B × 256 × 32 × 32	B × 128 × 64 × 64	128
Skip connection S2	Concatenate with E2	Channel-wise	128 + 128 channels	B × 256 × 64 × 64	256
Decoder block D2	Two Conv blocks	3 × 3/1	B × 256 × 64 × 64	B × 128 × 64 × 64	128
Decoder D1	Transposed convolution	2 × 2/2	B × 128 × 64 × 64	B × 64 × 128 × 128	64
Skip connection S1	Concatenate with E1	Channel-wise	64 + 64 channels	B × 128 × 128 × 128	128
Decoder block D1	Two Conv blocks	3 × 3/1	B × 128 × 128 × 128	B × 64 × 128 × 128	64
Resolution restoration 1	Bilinear upsampling	Scale factor 2	B × 64 × 128 × 128	B × 64 × 256 × 256	64
Refinement block 1	Conv + BN + ReLU	3 × 3/1	B × 64 × 256 × 256	B × 32 × 256 × 256	32
Resolution restoration 2	Bilinear upsampling	Scale factor 2	B × 32 × 256 × 256	B × 32 × 512 × 512	32
Refinement block 2	Conv + BN + ReLU	3 × 3/1	B × 32 × 512 × 512	B × 16 × 512 × 512	16
Segmentation output	Final convolution	1 × 1/1	B × 16 × 512 × 512	B × $C_{seg}$ × 512 × 512	$C_{seg}$

## References

[1] Zhang S., Zhang X., Wan S., Ren W., Zhao L., Shen L. (2024). Generative Adversarial and Self-Supervised Dehazing Network. IEEE Trans. Ind. Inform..

[2] Kutlimuratov A., Eshmurodov D., Tulaganova F., Utegenov A., Allayarov P., Khamzaev J., Saymanov I., Makhmudov F. (2026). Early Diagnosis of Blood Disorders via Enhanced Image Preprocessing and Deep Learning Modeling. BioMedInformatics.

[3] Buriboev A.S., Abduvaitov A., Jeon H.S. (2025). Integrating Color and Contour Analysis with Deep Learning for Robust Fire and Smoke Detection. Sensors.

[4] Abdusalomov A., Umirzakova S., Tashev K., Sevinov J., Temirov Z., Muminov B., Buriboev A., Safarova Ulmasovna L., Lee C. (2025). AI-Driven Boost in Detection Accuracy for Agricultural Fire Monitoring. Fire.

[5] Buriboev A., Muminov A. (2022). Computer State Evaluation Using Adaptive Neuro-Fuzzy Inference Systems. Sensors.

[6] Abdusalomov A., Umirzakova S., Bekmirzaev O., Dauletov A., Buriboev A., Kutlimuratov A., Nishanov A., Nasimov R., Oh R. (2025). From Anatomy to Genomics Using a Multi-Task Deep Learning Approach for Comprehensive Glioma Profiling. Bioengineering.

[7] Shavkatovich Buriboev A., Abduvaitov A., Jeon H.S. (2025). Binary Classification of Pneumonia in Chest X-Ray Images Using Modified Contrast-Limited Adaptive Histogram Equalization Algorithm. Sensors.

[8] Nishanov A., Mengturayev F., Ollamberganov F., Allayarov U., Khasanova M., Doniyorova G. (2026). An algorithm for creating a semi-synthetic dataset for diabetes. J. Math. Mech. Comput. Sci..

[9] Rasool M.J.A., Abdusalomov A., Kutlimuratov A., Ahamed M.J.A., Mirzakhalilov S., Shavkatovich Buriboev A., Jeon H.S. (2025). PixMed-Enhancer: An Efficient Approach for Medical Image Augmentation. Bioengineering.

[10] Nishanov A.K., Gulomjon Primovich D., Khasanova M.A. (2019). Improved algorithms for calculating evaluations in processing medical data. Compusoft.

[11] Khasanovich N.A., Bakirovich A.B., Primovich J.G., Akhramovna K.M., Khamdamjanovna M.M., Fakhriyevna U.Z. (2021). The algorithm for selection of symptom complex of ischemic heart diseases based on flexible search. J. Cardiovasc. Dis. Res..

[12] Nishanov A.K., Turakulov A.K., Turakhanov K.V. (1999). A decisive rule in classifying diseases of the visual system. Meditsinskaia Tekhnika.

[13] Nishanov A.K., Turakulov K.A., Turakhanov K.V. (1999). A decision rule for identification of eye pathologies. Biomed. Eng..

[14] An Q., Rahman S., Zhou J., Kang J. (2024). A Deep Convolutional Neural Network for Pneumonia Detection in X-ray Images with Attention Ensemble. Diagnostics.

[15] Kayumov M., Razzokov J., Makhkamov M., Radjabov M., Mukhamedov N., Khakimov M., Asrorov A.M., Khasanov O., Yashinov A., Tashmukhamedov M. (2025). 3CL^pro^ of SARS-CoV-2 as a new target for bufadienolides: In silico and in vitro study. J. Comput. Aided Mol. Des..

[16] Oltu B., Güney S., Yuksel S.E., Dengiz B. (2025). Automated Classification of Chest X-rays: A Deep Learning Approach with Attention Mechanisms. BMC Med. Imaging.

[17] Gopal S., Garg S., Riyazuddin M., Rachapudi V., Makharov K., Smerat A., Karimi R. (2026). NeuroExplain-net for transparent multi-class lung cancer screening using computed tomography. Intell.-Based Med..

[18] Zhang K., Liang W., Cao P., Mao Z., Yang J., Zaiane O.R. (2025). CorLabelNet: A comprehensive framework for multi-label chest X-ray image classification with correlation guided discriminant feature learning and oversampling. Med. Biol. Eng. Comput..

[19] Xu Q., Duan W. (2024). DualAttNet: Synergistic Fusion of Image-Level and Fine-Grained Attention for Multi-Label Lesion Detection in Chest Radiographs. Med. Image Anal..

[20] Ou C.-Y., Chen I.-Y., Chang H.-T., Wei C.-Y., Li D.-Y., Chen Y.-K., Chang C.-Y. (2024). Deep Learning-Based Classification and Semantic Segmentation of Tuberculosis Lesions in Chest X-ray Images. Diagnostics.

[21] Gopatoti A., Jayakumar R., Billa P., Patteeswaran V. (2024). DDA-SSNets: Dual Decoder Attention-Based Semantic Segmentation Networks for Chest X-ray Analysis. Biomed. Signal Process. Control.

[22] Miah M.A.I., Paul S., Das S., Hashem M.M.A. (2024). InfLocNet: Enhanced Lung Infection Localization and Disease Detection from Chest X-ray Images Using Lightweight Deep Learning. arXiv.

[23] Yoo R.E., Choi S.H. (2024). Deep Learning-Based Image Enhancement Techniques for Medical Imaging. Korean J. Radiol..

[24] Xu G., Zhang J., Wang L., Chen M., Liu X. (2024). SSP-Net: A Siamese-Based Structure-Preserving Network for Medical Image Enhancement. Med. Image Anal..

[25] Ye Z., Wang J., Liu H., Chen Y., Zhang X. (2024). Deep Learning-Based Cystoscopy Image Enhancement. Int. J. Comput. Assist. Radiol. Surg..

[26] Steinmetz S., Keller A., Braun M., Hoffmann J., Weber L. (2024). Impact of Deep Learning-Enhanced Contrast on Diagnostic Accuracy in Poorly Contrasted CT Angiography. Eur. J. Radiol..

[27] Park Y.W., Yoo R.-E., Shin I., Jeon Y.H., Singh K.P., Lee M.D., Kim S., Yang K., Jeong G., Ryu L. (2025). A Multinational Study of Deep Learning-Based Image Enhancement for Multiparametric Glioma MRI. Sci. Rep..

[28] Sharif S.M.A., Naqvi R.A., Biswas M., Loh W.-K. (2025). Deep Perceptual Enhancement for Medical Image Analysis. IEEE J. Biomed. Health Inform..

[29] Abduvaitov A., Buriboev A.S., Sultanov D., Buriboev S., Yusupov O., Kilichev J., Choi J.A. (2025). Enhancing Medical Image Segmentation and Classification Using a Fuzzy-Driven Method. Sensors.

[30] Ji Z., Zhang L., Li H., Zhao Y., Wang C., Liu M. (2024). ASD-Net: A U-Net-Based Asymmetric Spatial–Channel Convolution Network for Precise Kidney and Kidney-Tumor Segmentation. Med. Biol. Eng. Comput..

[31] Yao N., Hu H., Han C., Nan J., Li Y., Zhu F. (2024). An Automated Two-Stage Approach to Kidney and Tumor Segmentation from CT Images. Comput. Biol. Med..

[32] Hild O., Berriet P., Nallet J., Salvi L., Lenoir M., Henriet J., Thiran J.-P., Auber F., Chaussy Y. (2024). Automation of Wilms’ Tumor and Kidney Segmentation by Artificial Intelligence. Pediatr. Radiol..

[33] Al-Battal A.F., Tang V.H., Tran Q.D., Truong S.Q.H., Phan C., Nguyen T.Q., An C. (2024). Enhancing Lesion Detection in Liver and Kidney CT Scans Using Selective Deep Ensemble Segmentation. Comput. Biol. Med..

[34] Hao Z., Chapman B.E. (2025). Deep Learning-Based Cascade 3D Kidney Segmentation for Automated Renal Tumor Analysis. Abdom. Radiol..

[35] Özbay E., Özbay F.A., Gharehchopogh F.S. (2024). Kidney Tumor Classification on CT Images Using Self-Supervised Learning. Comput. Biol. Med..

[36] Feng X., Zhang Q., Yang Y., He H., Yang L., Zhao T., Li Q., Yang L., Gu Y., Chen Q. (2025). End-to-End Deep Learning for Diagnosis of Retroperitoneal Neoplasms. Eur. Radiol..

[37] Nizam M.B., Zlateva M., Davis J. (2024). J-CaPA: Joint Channel and Pyramid Attention Improves Medical Image Segmentation. arXiv.

[38] Liu J., Yang H., Zhou H.-Y., Yu L., Liang Y., Yu Y., Zhang S., Zheng H., Wang S. (2025). Swin-UMamba: Adapting Mamba-Based Vision Foundation Models for Medical Image Segmentation. IEEE Trans. Med. Imaging.

[39] Zhong X., Lu G., Li H. (2025). Vision Mamba and xLSTM-UNet for Medical Image Segmentation. Biomed. Signal Process. Control.

[40] Fan C., Yu H., Huang Y., Wang L., Yang Z., Jia X. (2025). SliceMamba with Neural Architecture Search for Medical Image Segmentation. Med. Image Anal..

[41] Liu Y., Feng Y., Cheng J., Zhan H., Zhu Z. (2025). MambaDiff: Mamba-Enhanced Diffusion Model for 3D Medical Image Segmentation. IEEE Trans. Image Process..

[42] Cheng Z., Guo J., Zhang J., Qi L., Zhou L., Shi Y., Ga Y. (2025). A Mamba-Based Framework with Global-to-Local Modeling for Medical Image Segmentation. IEEE Trans. Med. Imaging.

[43] Nguyen T.-N.-Q., Ho Q.-H., Nguyen V.Q., Pham V.-T., Tran T.-T. (2025). ADC-MambaNet: A Lightweight U-Shaped Architecture for Medical Image Segmentation. Comput. Methods Programs Biomed..

[44] Lu Y., Sun F., Wang J., Yu K. (2025). Automatic Joint Segmentation and Classification of Breast Ultrasound Images via Multi-Task Learning with Object Contextual Attention. Front. Oncol..

[45] He Q., Yang Q., Su H., Wang Y. (2024). Multi-Task Learning for Segmentation and Classification of Breast Tumors from Ultrasound Images. Comput. Biol. Med..

